# Do oral contraceptives affect young women’s memory? Dopamine-dependent working memory is influenced by COMT genotype, but not time of pill ingestion

**DOI:** 10.1371/journal.pone.0252807

**Published:** 2021-06-10

**Authors:** Laura Gravelsins, Katherine Duncan, Gillian Einstein

**Affiliations:** 1 Department of Psychology, University of Toronto, Toronto, Ontario, Canada; 2 Rotman Research Institute, Baycrest Hospital, Toronto, Ontario, Canada; 3 Dalla Lana School of Public Health, University of Toronto, Toronto, Ontario, Canada; 4 Center for Medical Image Science (CMIV) at Linköping University, Linköping, Östergötland, Sweden; 5 Department of Gender Studies, Linköping University, Linköping, Östergötland, Sweden; University of Insubria, ITALY

## Abstract

**Background:**

Despite the widespread use of oral contraceptives (OCs), and the well-documented influence of estrogens, notably 17β-estradiol (E2), on cognition, research relating OCs to working memory is limited and mixed. Two factors may contribute to these mixed findings: 1) pharmacokinetics of oral contraceptives, which drive fluctuations in synthetic hormone levels; and 2) genetic polymorphisms related to dopamine degradation and working memory, which interact with E2. This research investigated whether the pharmacokinetics of oral contraceptives, in concert with the single nucleotide polymorphism (*Val*^*158*^*Met*; rs4680) of the catechol-o-methyltransferase gene (*COMT*), influence working memory performance.

**Methods:**

University-age women taking and not taking OCs were tested for working memory and genotyped for *COMT*. If they were not taking OCs (n = 62), sessions occurred in the early follicular (low E2) and late follicular (high E2) phase. If they were taking OCs (n = 52), sessions occurred 1–2 hours after (high ethinyl estradiol, EE) and ~24 hours after (low EE) pill ingestion. Working memory was tested using the N-back, AX-CPT, Digit Span, and Digit Ordering Tasks. Data were analyzed using multilevel models with estrogen condition, *COMT*, and group as predictors, controlling for mood and practice effects.

**Results:**

For women taking OCs, time of pill ingestion did not influence performance. However, the subgroup with *COMT val/val* (low dopamine) were less accurate on 2-back lure trials than those with *COMT met/met* (high dopamine). For women not taking OCs, cycle phase moderated *COMT*’s influence on lure accuracy. When compared, women taking OCs had higher AX-CPT proactive control indices than those not taking OCs.

**Conclusion:**

These findings suggest that oral contraceptives are not detrimental for young women’s working memory and that they may increase proactive control. The more pronounced effects of *COMT* in women taking OCs suggests that, in women taking OCs, suppressed endogenous E2–not fluctuating EE levels–may be more relevant for working memory. Future studies are needed to differentiate effects of endogenous versus synthetic estrogens on working memory.

## Introduction

Oral contraceptives (OCs), a widely used form of hormonal birth control, are also often prescribed for treating endometriosis and polycystic ovarian syndrome, and managing symptoms related to the menstrual cycle [1,2]. From 2007–2011, approximately 1.3 million women in Canada took OCs every month [3], and from 2015–2017 approximately 9.1 million women in the United States took OCs [4]. Despite the widespread use of the pill, surprisingly little is known about its effects on women’s cognition.

Indeed, there are many reasons to expect OCs to influence women’s cognition, as OCs contain synthetic estrogen, and ample evidence suggests estrogens influence the brain and behaviour. For example, 17β-estradiol (E2) binds to and activates estrogen receptors throughout the brain, influencing neurotransmission, gene transcription and neurogenesis [5]. Accordingly, E2 has known effects on cognition, particularly memory. Verbal memory performance, for instance, improves during menstrual cycle phases with higher E2 [6], while visuospatial performance improves during lower E2 phases [7]. Women with ovarian removal prior to natural menopause are at greater risk for developing dementia [8], but show preserved performance on associative memory and episodic memory [9] as well as working memory [10] with E2 replacement. Likewise, premenopausal women with pharmacologically induced E2 suppression show working memory deficits [11]. With these pronounced effects of E2 on women’s memory, it stands to reason that pharmacologically influencing estrogen with OCs could likewise influence women’s memory.

While research comparing women taking OCs (OC) to those naturally cycling and not taking OCs (NOC) is sparse, what exists in younger women shows a correlation of taking OCs with higher prefrontal cortical grey matter volume [12], and reduced resting-state connectivity within the executive control and default mode networks [13] as compared to NOC. This might suggest that OCs would also affect working memory, as it is mediated by these brain regions. However, data on the behavioural consequences of OCs on working memory and executive function are mixed. Some studies show impairments in OC as compared to NOC, while others show improvements or no effects (see [14,15] for review).

One factor that may contribute to these inconsistent findings are the pharmacokinetics of OCs which have not been considered in experimental design. Endogenous hormone levels in OCs are continually suppressed and are comparable to or lower than the levels in early follicular phase of NOC, the menstrual cycle phase associated with lowest circulating sex hormone levels (see [16] for review). By contrast, circulating levels of exogenous hormones vary substantially within a day. Levels of the main synthetic estrogen contained in OCs, ethinyl estradiol (EE), peak in the blood 1–2 hours after pill ingestion and then gradually decline, meaning EE levels are at their lowest right before the next pill is ingested [17]. Further, EE binds to the same estrogen receptors as E2 in the brain [18]. Although no study has considered variations in EE levels over a 24-hour period when investigating cognition in OC, one study found visuospatial performance was worse in women who have taken their pill for the day as compared to women who had not yet taken their pill [7]. This finding suggests that short term fluctuations in EE may influence cognitive performance. This initial positive finding highlights how studies that carefully consider time of pill ingestion are needed to better understand the effects of OC on working memory.

**Fig 1:**
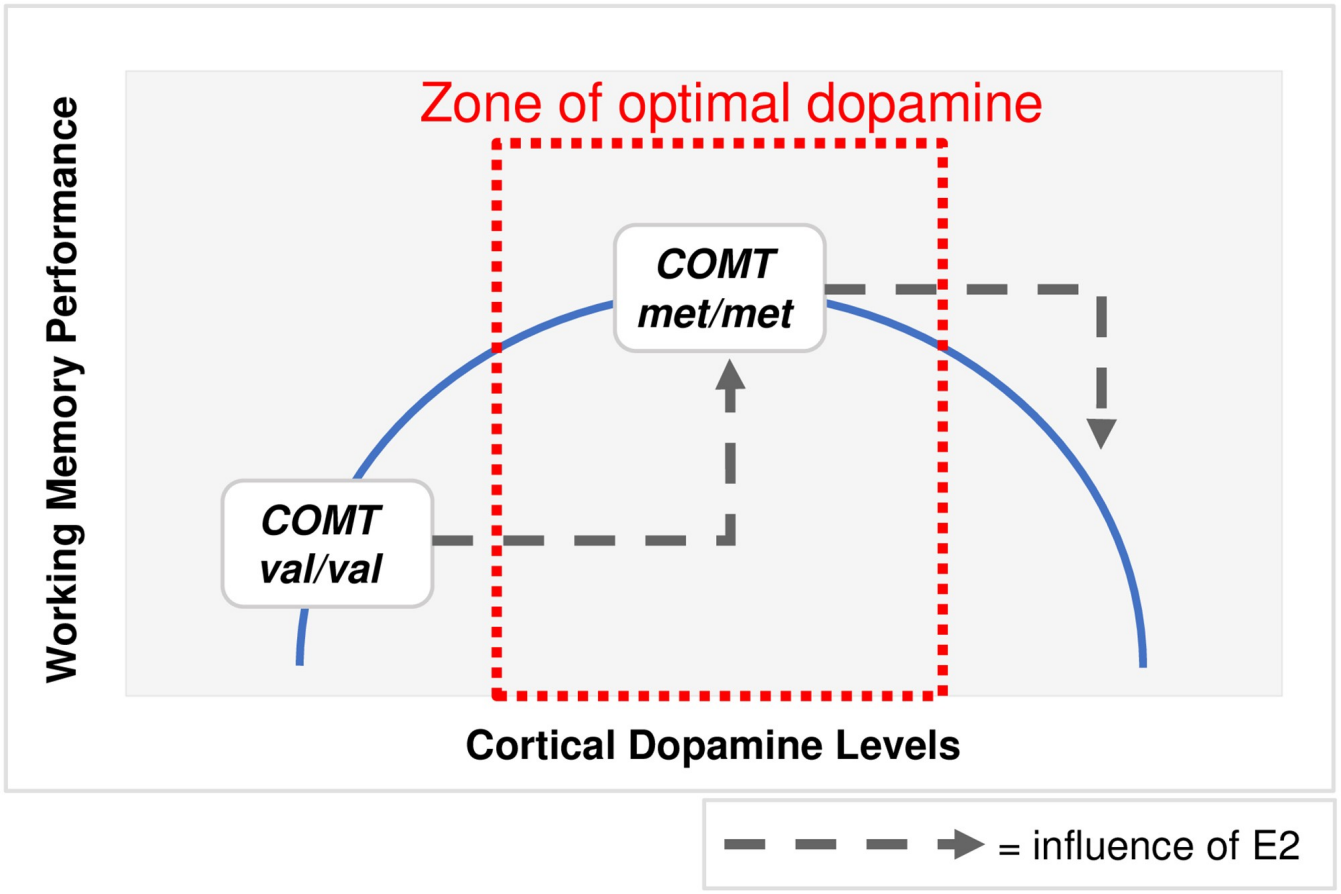
The inverted-U dopamine response curve and working memory. The x-axis reflects the levels of dopamine in the PFC, and the y-axis represents working memory performance. The “zone of optimal dopamine,” the level of dopamine in the PFC that would result in optimal working memory performance, is shown by the dashed red box. The position of women who are *COMT val/val* and *COMT met/met* are shown on the curve. In general, *met/met* outperform *val/val* on working memory tasks, which is attributable to their optimal levels of dopamine. The influence of high E2 (E2 levels characteristic of the late follicular phase) on *COMT val/val* and *COMT met/met* women is shown by the dashed grey arrows. High E2 is more advantageous for women with *COMT val/val* than women with *COMT met/met*, and the opposite is true for low E2 [22].

Also overlooked when considering OCs effect on cognition is their possible interaction with dopamine—a neurotransmitter that strongly modulates working memory. The relationship between dopamine and memory is complicated, with some research suggesting an inverted-U dopamine response curve, in which working memory performance is highest when dopamine levels are neither very high nor very low ([19]; Fig 1). Endogenous E2 may well be implicated in producing these optimal dopamine levels, with E2 acting as a dopaminergic agonist [20]. One study showed that a single nucleotide polymorphism (SNP, *Val*^*158*^*Met*; rs4680) of the gene coding for catechol-o-methyltransferase (*COMT*), the protein that degrades dopamine at synapses in the prefrontal cortex (PFC; [21]), interacts with E2 levels to mediate working memory performance [22]. The findings from this study suggest that the interaction between prefrontal dopamine and E2 levels is complex, with E2 making a positive contribution to working memory when dopamine levels are low (*COMT val/val*) and a negative one when dopamine levels are high (*COMT met/met*). Thus, OC studies grouping women with different *COMT* genotypes and hence, dopamine levels, might mask high EE’s (or low E2’s) relationship to working memory.

Therefore, we asked the following questions: 1) do the pharmacokinetics of EE–as measured by pill ingestion time–influence OC’s working memory?; and 2) does EE interact with dopamine–as measured by *COMT* genotype–to affect working memory? We hypothesized that EE level (i.e. pill time) would influence cognitive performance. We also hypothesized that, if OCs have the same effect on working memory as endogenous E2, higher EE 1–2 hours after pill ingestion would improve working memory in low dopamine conditions (*COMT val/val*) and reduce it in high dopamine conditions (*COMT met/met*). On the other hand, since endogenous E2 remains stably low in women taking OCs [16,23–25], an alternative possibility would be that low E2 levels would drive OC with high dopamine (*COMT met/met*) to outperform OC with low dopamine (*COMT val/val*), independent of EE levels.

## Material and methods

### Participants

This study was approved by the University of Toronto Social Sciences, Humanities and Education Research Ethics Board (Research Information System Protocol Number: 35242) and was in accordance with the declaration of Helsinki. Women ages 18–30 were recruited through the University of Toronto’s Introductory Psychology course and community advertisements. Potential participants were phone screened prior to study enrolment and provided written informed consent prior to testing.

**Fig 2:**
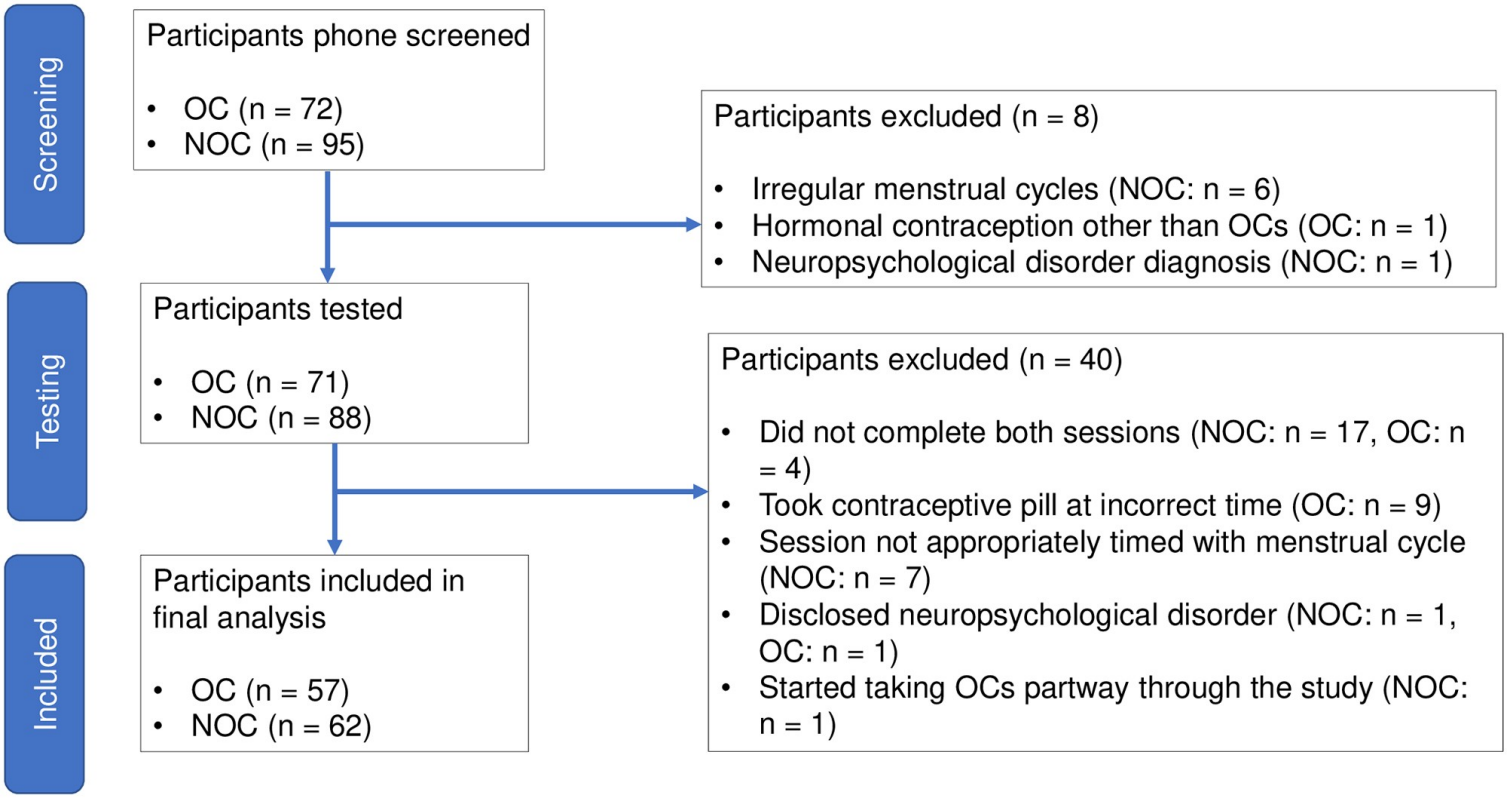
Flow diagram of the participant exclusion process.

Exclusion criteria were: not fluent in English, currently pregnant or pregnant within the past 6 months, history of neurological or psychiatric disorders including concussion with loss of consciousness, regular cigarette smoking, currently taking psychoactive medications or exogenous hormones (other than OCs), and a diagnosis of an endocrine disorder. Inclusion criteria were: for NOC, not using hormonal contraception, and an average menstrual cycle length of 21–35 days, with regular menstrual cycles for the past 6 months, and for OC, use of a combined pill for at least 3 consecutive months prior to participation. Type of synthetic hormones in the OCs was neither an exclusion nor an inclusion criterion. Initially, 167 participants (95 NOC and 72 OC) were recruited for our study. After exclusions, the final cohort had 119 participants: 62 NOC and 57 OC (Fig 2). Since the majority of our recruitment was done through a large Psychology course using a credit system, and the experiment required two in-person visits, testing at the appropriate menstrual cycle phase, and testing at the appropriate pill ingestion time, our busy undergraduates did not always comply.

### Procedure

We used a within-subjects design. Each person participated in two testing sessions, timed according to their menstrual cycle phase (NOC) or pill ingestion (OC). For NOC, one session occurred during the early follicular phase (cycle day 1–5) and another during the late follicular phase (cycle day 9–14), determined by forward counting from day of menstruation, and when possible, referencing period tracking applications. OC were scheduled 1–2 hours after pill ingestion and again, roughly 2 weeks later approximately 24 hours after pill ingestion. The order of sessions was counterbalanced for both groups.

Participants were tested from 10:30am to 6:00pm, an optimal time of day for younger adults to undergo cognitive assessment [26]. Participants were administered a demographics questionnaire to obtain information on their age, ethnicity, education, type of OC, and reproductive history. Mood at time of testing was determined by administering the Centre for Epidemiologic Studies Depression scale (CES-D; [27]). Subsequently, participants completed a series of working memory tasks, and provided a saliva sample for genotyping the *COMT Val*^*158*^*Met* SNP.

### Tasks

#### N-back

This task was chosen because it tests verbal working memory [28]. It was the primary test of dopamine-dependent working memory because it was previously used to demonstrate that NOC performance depended on the interaction between dopamine levels (*COMT Val*^*158*^*Met* SNP) and E2 as measured in blood [22]. Additionally, it engages the dorsal lateral PFC [29], and requires continual maintenance and updating of the letter sequence, and inhibition of responses to distracting lure trials.

Participants completed a computerized 1-back and 2-back task with letter stimuli using Inquisit 5 software (Millsecond, Ltd). Prior to data collection, participants completed a practice 1-back and 2-back block. Each letter was presented for 1000 ms followed by a 1000 ms fixation cross. Participants were instructed to make a button press for target trials when the current letter was the same as ‘N’-positions back in the sequence (N = 1 in the 1-back, N = 2 in the 2-back). A different button response was required for non-target trials. Consistent with others, the 2-back condition included hard-to-reject lure trials that were either a 1-back or 3-back match to the current stimulus [22]. In the 1-back task, ~81% of trials displayed non-targets and ~19% displayed targets. In the 2-back, ~19% of trials displayed targets, ~18% lures, and ~63% non-targets. In total, participants completed 5 N-back blocks (two 1-back and three 2- back), each consisting of 32 trials. Reaction time and accuracy of responses were recorded.

#### Forward and backward digit span (DS)

This task was selected because it also tests verbal working memory. In the Forward DS, participants were instructed to repeat back sequences of digits read aloud by the experimenter; in the Backward DS, participants were asked to repeat the sequence in reverse order. There were 2 trials for each sequence length, and the maximum sequence length was 8 digits on the Forward task and 7 digits on the Backward task [30]. The Forward DS assesses the passive retention of information. Thus, it was used as a control task for the Backward DS, which requires participants to maintain and manipulate (reorder) the sequence of numbers.

#### Digit ordering task

This task was selected because it requires working memory and engages the dorsolateral PFC [31]; it requires participants to maintain and continually update their mental representation of numbers. Participants were instructed to say aloud the numbers 1 to 10 in a random order, without repeating or omitting numbers and avoiding the use of patterns (i.e. saying odds then evens, or saying the highest then lowest numbers). They were instructed to tell the experimenter when they thought they had said all 10 numbers and to begin a new set when ready. The total number of repetitions and omissions (i.e., total working memory errors) from the 10 trials were recorded. Using patterns results in fewer working memory errors on this task. If participants used patterns for more than 3 trials, their data were excluded. For individuals with 1 to 3 trials with patterns, the pattern trials were discarded, and a weighted total for the number of working memory errors was calculated (total working memory errors/number of usable trials X 10).

#### AX-continuous performance task (AX-CPT)

This task was selected because it requires the maintenance of goal-relevant information and inhibition of prepotent responses, important components of working memory [32], and because task performance is sensitive to dopamine levels [33,34]. A computerized AX-CPT on Inquisit 5 software (Millsecond, Ltd) was used. Participants were shown a sequence of letters, and each letter was presented for 300 ms followed by a 1200 ms fixation cross. A target trial consisted of a valid cue, the letter ‘A’, followed by a valid probe, the letter ‘X’. Non-target trials consisted of a valid cue followed by an invalid probe, termed “AY” trials, an invalid cue followed by a valid probe, termed “BX” trials, or an invalid cue followed by an invalid probe, termed “BY” trials. An on-screen prompt reminded participants to make a button response during probe presentation. A different button response was required for target and non-target trials.

Participants completed 2 blocks, each consisting of 150 trials, of which 70% were AX, 10% were AY, 10% were BX, and 10% were BY. A high proportion of AX trials was used to create a prepotent response tendency to AX. The reaction time and accuracy of responses were recorded.

### Statistical analysis

All analyses were conducted in R 3.6.1 [35]. For the N-back, trial-by-trial performance on 2-back lure trials were our primary focus as they had previously been shown to be sensitive to *COMT* genotype and E2 levels [22]. Trial-by-trial accuracy was modeled as a function of *COMT*, group (NOC or OC), estrogen condition, and their interactions. “Estrogen condition” referred to cycle phase (early follicular and late follicular, a proxy for low and high endogenous estradiol levels respectively) in NOC and to pill ingestion time (24 vs 1–2 hours since pill ingestion, a proxy for low and high EE levels respectively) in OC. Random slopes were modelled for estrogen condition, grouped by participant, and for each participant a random intercept was modelled. Session and estrogen condition were partially correlated within participant, so session was only included as a covariate to control for practice effects and not a random effect. CES-D score was also a covariate in order to control for mood, as premenstrual dysphoric disorder, although only in a minority of women, was not an exclusionary criterion, and use of OCs has been associated with first diagnosis of depression and increased use of antidepressants [36]. The model was estimated with an unstructured covariance matrix using the glmer function with a binomial linking function from the lme4 package [37].

Trial-by-trial reaction time on correct trials was modeled in the same way using the lmer function from the lme4 package [37]. However, to limit the influence of outliers, reaction times 2.5 standard deviations above or below each participant’s mean were trimmed. As at least 100 ms is needed for stimulus perception and motor response selection, reaction times ≤100ms were discarded. Furthermore, reaction times were log transformed so that residuals better approximated a normal distribution.

If the full models for trial-by-trial performance yielded significant effects, reduced models were run separating NOC from OC to understand the source of the effect. Additional exploratory analyses were run examining trial-by-trial accuracy and reaction time on target and non-target trials in the 1-back and 2-back tasks. The False Discovery Rate (FDR) correction for multiple comparisons was applied to exploratory analyses using the p.adjust function from the stats package.

For the AX-CPT, a proactive behavioural index (PBI) was calculated to quantify the extent of proactive control (planning to respond after seeing an A) vs. reactive control (considering responding after seeing an X) used by participants [38]. The computation for PBI accuracy and PBI reaction time on correct trials, respectively, is as follows: (-1)*(AY–BX)/(AY + BX), and (AY–BX)/(AY + BX). Proactive control is expected to result in lower accuracy on AY trials than BX trials, and longer response times on AY trials than BX trials. Reactive control is expected to have the reverse pattern. Thus, a positive PBI reflects greater proactive control.

AX-CPT PBIs, number of errors on the Digit Ordering Task, and maximum number of digits on the DS Task were also analyzed using multilevel models. COMT, group, estrogen condition with their interactions were included as predictors, and CES-D and session as co-variates. A random intercept was modelled for each participant. Random slopes were not included because these models only contained one summary observation per estrogen condition per participant. Prior to calculating PBI reaction time, reaction times were trimmed using the same criteria as the N-back task and log transformed. The models were estimated with an unstructured covariance matrix using the lmer function from the lme4 package [37].

Six of the 119 eligible participants completed some, but not all of the tasks, and were therefore excluded from analysis of those tasks (NOC = 3, OC = 3; [39]). Sensitivity analyses at the end of recruitment revealed 80% power to detect group differences as small as Cohen’s *d* = 0.46 (0.27; observation-level effect sizes are in parentheses), estrogen condition differences as small as *d* = 0.27 (0.26), genetic differences as small as *d* = 0.71 (0.25), and interactions as small as *d* = 1.17 (0.26).

## Results

### Demographics

The average age of the 119 participants was 19.9 ± 0.2 years (±*SE*, range = 18–28), and the average CES-D score was 13.23 ± 0.77 (±*SE*, range = 0–47). The *COMT* distribution of the sample was in Hardy Weinberg equilibrium (χ2 = 4.03, p = .13), with 20 *met/met*, 57 *met/val*, and 42 *val/val*. There was no significant difference in age (Wilcoxon ranks sum test: *W* = 1859.5, *p* = .61), CES-D score (Wilcoxon ranks sum test: *W* = 1703.5, *p* = .74), or *COMT* distribution (Chi-square test: *χ2* = 5.05, *p* = .08) between NOC and OC (Table 1). There was a significant difference in the proportion of those who identified as Caucasian (Z-score test: *Z* = 3.50, *p* = 0.00046; Table 1).

**Table 1:** Demographics and *COMT* distribution of NOC and OC.

	NOC (N = 62)	OC (N = 57)
Age*(years, mean ± SE)	20.2 ± 0.4	19.7 ± 0.3
**Delay between sessions (days, mean ± SE)**	16.8 ± 11.1	16.7 ± 0.9
CES-D*(mean ± SE)	12.9 ± 1.1	13.6 ± 1.1
**Ethnicity (% Caucasian)**	19.30%	50%
**Genotype *Met/met* Count**^†^	7 (2)	13 (2)
**Genotype *Met/val* Count**^†^	28 (12)	29 (14)
**Genotype *Val/val* Count**^†^	27 (18)	15 (8)

*Reported age and CES-D scores are those at session 1.

^†^ The value in parentheses denotes the number of women who were tested at their low session first.

The majority of OCs being taken consisted of a monophasic pill formulation containing 20 ug of EE and 100 ug of levonorgestrel. EE was the synthetic estrogen in all OCs, and doses ranged from 20 to 35 μg. The progestin component was variable in type and dose (Table 2). Average pill duration was 2.11 ± 0.22 years (*±SE*, range = 0.33–8 years). NOC had an average menstrual cycle length of 28.51 ± 0.31 days (*±SE*, range = 22–34.5 days).

**Table 2:** Oral contraceptive pill formulations.

N	Synthetic Estrogen		Progestin			Brand names
	Name	Dose (mcg)	Name	Generation	Dose (mcg)	
32	Ethinyl Estradiol	20	Levonorgestrel	2nd	100	Alesse, Alysena, Aviane, Optilova
1	Ethinyl Estradiol	30, 10	Levonorgestrel	2nd	150	Seasonique
6	Ethinyl Estradiol	30	Desogestrel	3rd	150	Mirvala, Marvelon
1	Ethinyl Estradiol	20	Desogestrel	3rd	150	Mercilon
3	Ethinyl Estradiol	30	Drospirenone	4th	3000	Yasmin
4	Ethinyl Estradiol	35	Cyproterone Acetate	3rd	2000	Cyestra, Diane
2	Ethinyl Estradiol	10	Norethindrone Acetate	3rd	1000	Lo Lestrin
1	Ethinyl Estradiol	35	Norgestimate	3rd	180, 215, 250	Tricyclen
4	Ethinyl Estradiol	25	Norgestimate	3rd	180, 215, 250	Tricira Lo
3	Ethinyl Estradiol	35	Norgestimate	3rd	250	Cyclen

### N-back

#### Trial-by-trial lure 2-back accuracy

The full multilevel model for 2-back lure accuracy revealed *met/met* women were significantly more accurate than *val/val* women on lure trials (β = -0.47, *SE* = 0.20, *z* = -2.39, *p* = .017). Additionally, it showed significant 3-way interactions between *COMT*, estrogen condition, and group (*val/val* vs. *met/met*: β = .41, *SE* = .14, *z* = 2.94, *p* = .003; *met/val* vs. *met/met*: β = .38, *SE* = .13, *z* = 2.82, *p* = .005). Therefore, reduced models separating NOC from OC were run to understand gene-estrogen relations within each group.

**Fig 3:**
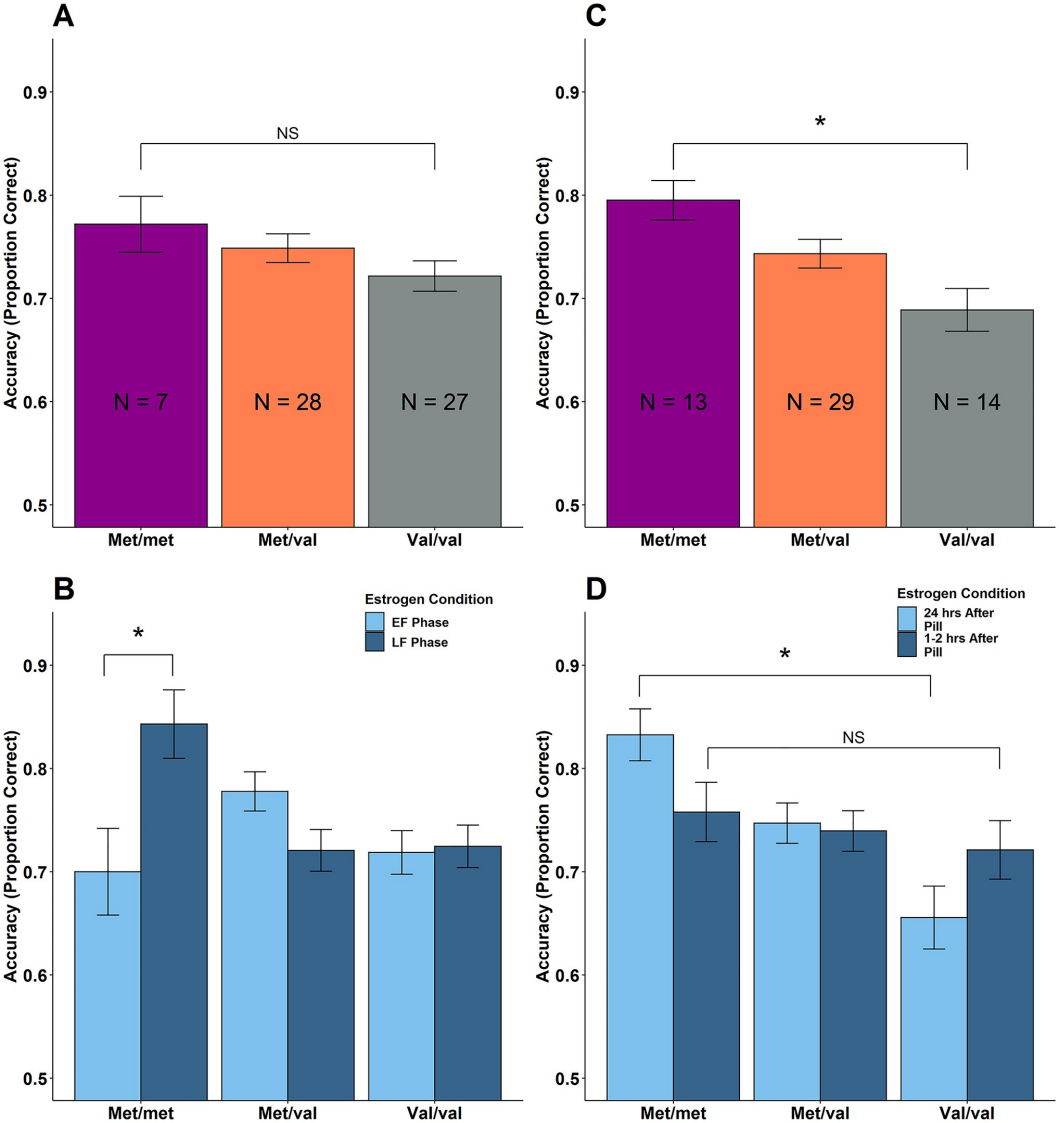
N-back lure accuracy is influenced by *COMT*, group and estrogen condition. In NOC, 2-back lure accuracy is related to the interaction of *COMT* and estrogen condition but in OC it is influenced by *COMT* alone. Reduced sample with only NOC: (A) 2-back lure accuracy was unaffected by *COMT* genotype; (B) *met/met* were significantly more accurate in the high estrogen condition (late follicular) than in the low estrogen condition (early follicular) phase, while *met/val* and *val/val* were less influenced by their estrogen condition. Reduced sample with only OC: (C) *met/met* had significantly higher accuracy on 2-back lures than *val/val*; (D) pill time was not a significant moderator of *COMT* on 2-back lure accuracy, however, differences between *met/met* and *val/val* were significant 24 hours, but not 1–2 hours, after pill ingestion. Error bar refers to SEM, *p<0.05 Abbreviations: NS, not significant; N, sample size; EF, early follicular; LF, late follicular; hrs, hours.

The reduced model with NOC revealed no significant differences between *met/met* and *val/val* (β = -.34, *SE* = .31, *z* = -1.10, *p* = .27), or *met/met* and *met/val* (β = -.17, *SE* = .31, *z* = -.54, *p* = .59) on 2-back lure trial accuracy (Fig 3A). On the other hand, estrogen condition in NOC was a significant moderator of *COMT* on 2-back lure accuracy (Fig 3B), such that *met/met* were significantly more accurate in the high estrogen condition (late follicular) than in the low estrogen condition (early follicular) phase (β = .55, *SE* = .17, *z* = 3.17, *p* = .002); however, estrogen condition had less of an effect on *met/val* (β_interaction_ = -.65, *SE* = .18, *z* = -3.48, *p* = .0005) and *val/val* (β_interaction_ = -.55, *SE* = .19, *z* = -2.89, *p* = .004).

The reduced model with OC revealed *val/val* OC were significantly less accurate than *met/met* OC on 2-back lures (β = -0.60, *SE* = 0.24, *z* = -2.48, *p* = .013; Fig 3C); a similar, though not statistically significant advantage, was seen for *met/met* compared to *met/val* (β = -0.34, *SE* = 0.21, *z* = -1.58, *p* = .11; Fig 3C). However, estrogen condition (i.e. pill ingestion time) did not significantly influence lure accuracy (β = -.02, *SE* = .07, *z* = -.03, *p* = .78; Fig 3D) or moderate the effects of *COMT* on 2-back lure accuracy in OC (β_interaction_ = .16, *SE* = .11, *z* = 1.47, *p* = .14; Fig 3D).

In summary, NOC performance showed an interaction of *COMT* and E2 condition but were unaffected by *COMT* alone. On the other hand, OC performance on the 2-back lure was not related to time of pill ingestion but was related to *COMT* condition.

#### Trial-by-trial lure 2-back reaction time on correct responses

The multilevel models for 2-back lure reaction times revealed no significant effects of or interactions between *COMT*, whether or not OCs are taken, and estrogen condition (for nonsignificant models, the effect with the lowest p-value is provided; *met/val* vs. *met/met*: β = -.03, *SE* = .02, *t* = -1.23, *p* = .22).

#### N-back exploratory analyses

None of the 1-back or 2-back task exploratory analyses met the alpha threshold following the liberal FDR correction. Results for the exploratory analyses are reported in the supporting information.

### Forward/backward DS task and digit ordering task

The multilevel model for the Forward DS revealed a trending 3-way interaction between *COMT*, estrogen condition, and group (*val/val* vs. *met/met*: β = .26, *SE* = .15, *t* = 1.69, *p* = .09). Therefore, reduced models separating NOC from OC were run.

**Fig 4:**
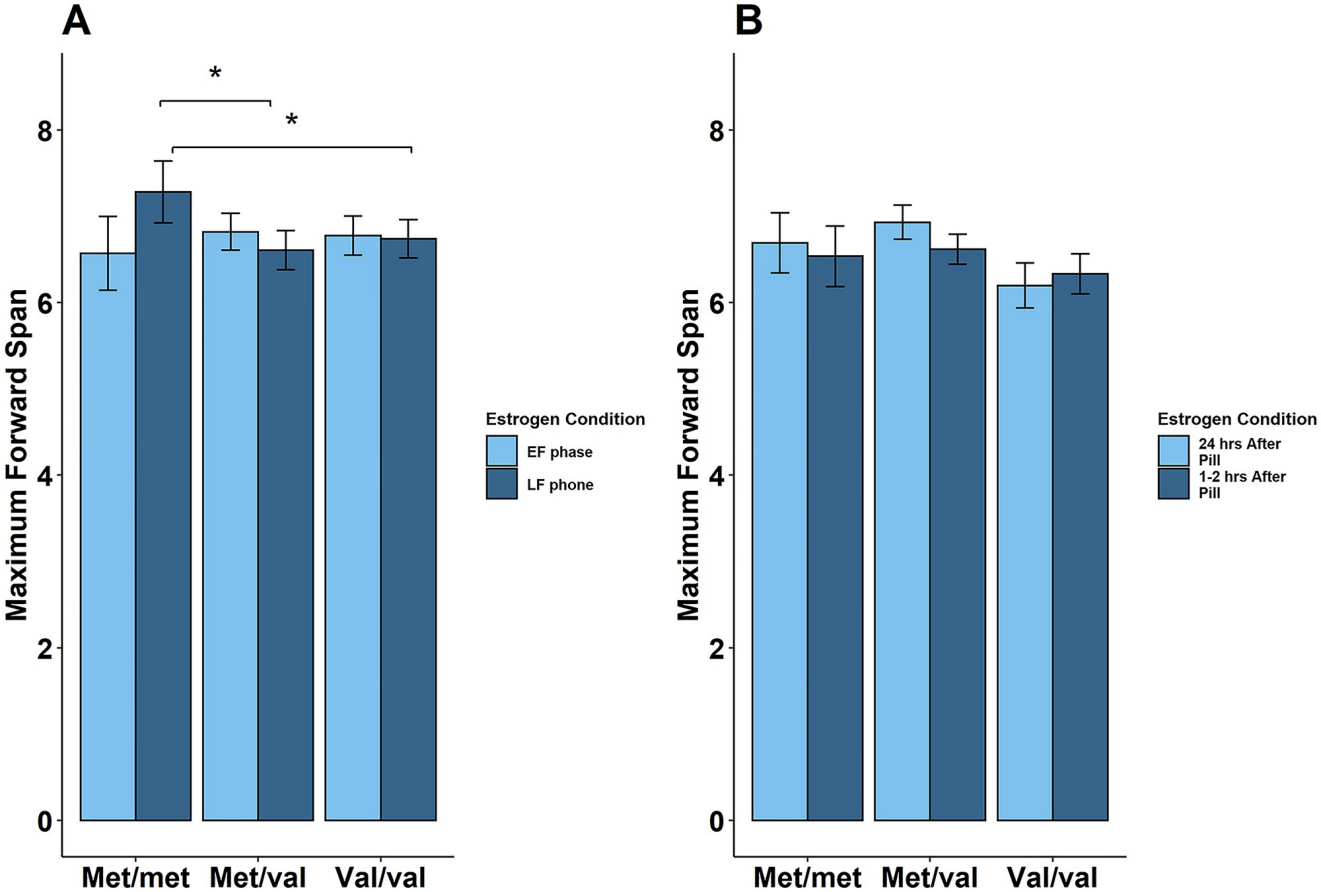
Estrogen condition interacts with *COMT* in the forward digit span. (A) Reduced sample with only NOC: *Met/met* NOC remembered more digits in the late follicular phase than *met/val* and *val/val*. (B) Reduced sample with only OC: No effect or interactions between EE condition and *COMT* were found. Error bars refer to SEM, *p<0.05 Abbreviations: EF, early follicular, LF, late follicular, EE, ethinyl estradiol, hrs, hours.

The reduced model with NOC did reveal that estrogen condition was a trending moderator of *COMT* on number of digits remembered (β = .41, *SE* = .21, *t* = 2.00, *p* = .05; Fig 4A), such that *met/met* tended to be more accurate in the high estrogen condition (late follicular) than the low estrogen condition (early follicular) phase, but *met/val* (β_interaction_ = -.51, *SE* = .23, *t* = -2.20, *p* = .03) and *val/val* (β_interaction_ = -.51, *SE* = .24, *t* = -2.14, *p* = .04) were comparatively less influenced by estrogen condition. On the other hand, the reduced model with OC revealed no significant effects or interactions between *COMT* and estrogen condition (estrogen condition: β = .25, *SE* = .14, *t* = 1.81, *p* = .07; Fig 4B).

There were no significant effects or interactions between *COMT*, group, and estrogen condition in the multilevel models for maximum span in the Backward DS (estrogen condition: β = .25, *SE* = .14, *t* = 1.81, *p* = .07) or total working memory errors on the Digit Ordering Task (3-way interaction between *COMT*, estrogen condition, and group; *val/val* vs. *met/met*: β = 1.42, *SE* = .86, *t* = 1.64, *p* = .10).

### AX-CPT

**Fig 5:**
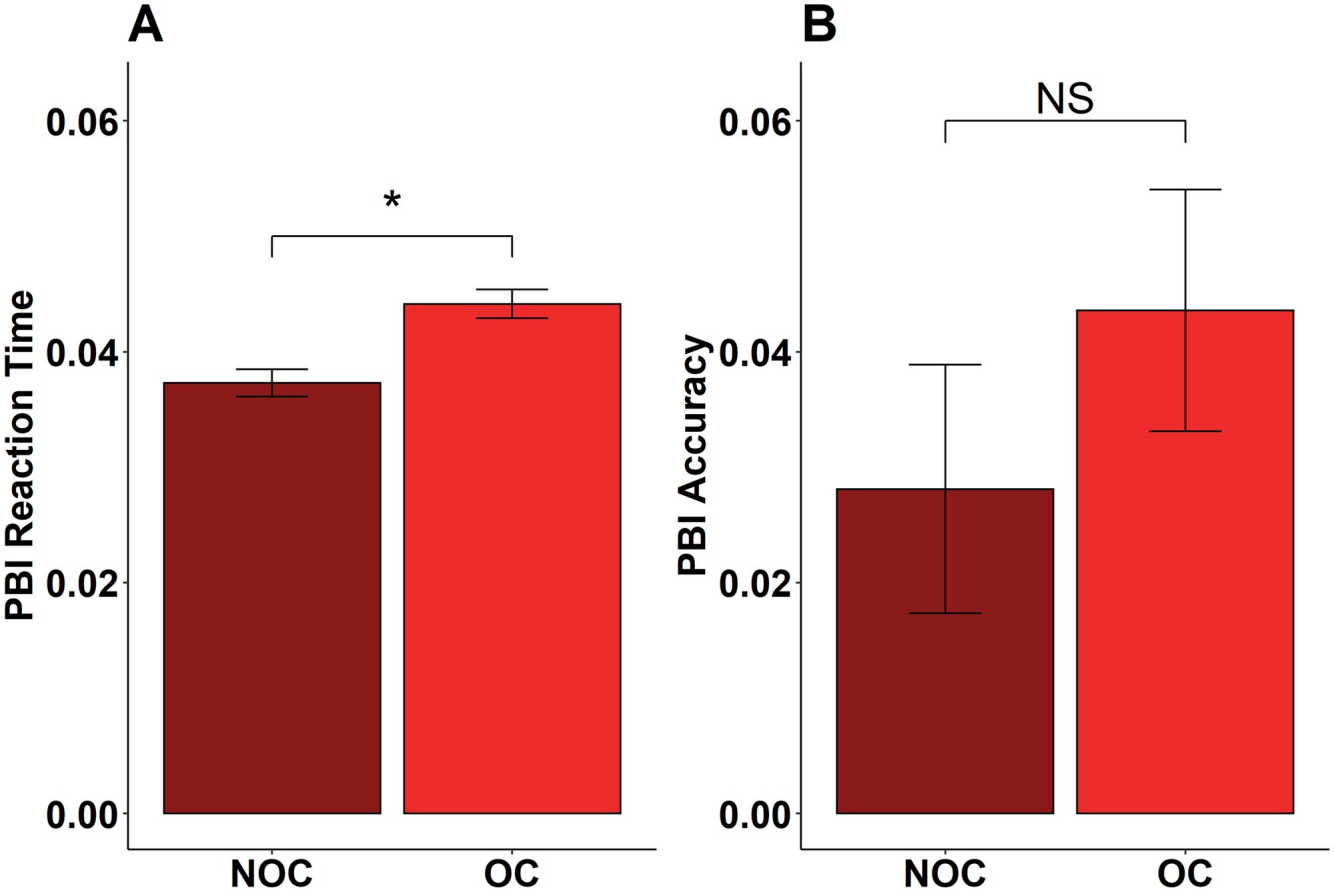
PBI reaction time depends on group. (A) NOC have significantly lower PBI reaction time than OC; (B) PBI accuracy is comparable between NOC and OC. Error bars refer to SEM, *p<0.05, NS = not significant.

The multilevel model for PBI reaction time on correct trials revealed a significant effect of group, such that NOC had a significantly lower PBI reaction time (i.e. a lower proactive control score) than OC (β = .004, *SE* = .001, *t* = 3.33, *p* = .001; Fig 5A). Otherwise, there was no influence of *COMT* or estrogen condition on PBI reaction time. The multilevel model for PBI accuracy showed no significant effects of or interactions between *COMT*, group, and estrogen condition (β = -.03, *SE* = .02, *t* = -1.23, *p* = .22; Fig 5B).

## Discussion

The two goals of our study were to determine whether the pharmacokinetics—time of OC pill ingestion—influences women’s working memory, and whether synthetic estrogens, like endogenous estrogens, interact with dopamine to affect working memory. To our knowledge, this is the first study to determine whether time of contraceptive pill ingestion affects any type of cognition and whether there might be an interaction between dopamine levels and EE.

### Pill ingestion time

We hypothesized that pill ingestion time would have an effect on working memory on its own, and that it would also modulate the influence of *COMT* on working memory. If this had been the case, we would have observed a difference in working memory performance in OC between 1–2 hours and 24 hours after their pill ingestion. We would have also observed improved working memory performance with higher EE (1–2 hours after pill ingestion) in low dopamine conditions (*COMT val/val*) and reduce it in high dopamine conditions (*COMT met/met*). However, we observed no difference in performance between these two proxies for EE levels. Since there was no fluctuation in working memory performance depending on the time of pill ingestion (on its own or in conjunction with *COMT* genotype), our results suggest that there are no EE effects on working memory.

### COMT genotype and N-back performance

In OC, we found a direct effect of *COMT* such that *met/met* had significantly higher accuracy on the 2-back lures than *val/val*. This finding, compounded with the fact that pill ingestion time did not affect performance, suggests that endogenous estrogens, not exogenous EE, likely mediates working memory performance in OC. OCs suppress ovarian hormone production, such that their levels are comparable to or fall below the range of E2 levels in the early follicular phase of NOC (see [16] for review), and remain low unless active pills are stopped for several days [25]. Thus, the suppressive action of OCs on endogenous E2 may disproportionately benefit working memory in those who have higher PFC dopamine concentrations.

Consistent with this possibility, in OC, we found that 2-back lure accuracy only showed a significant relationship with *COMT* 24 hours after pill ingestion. This is consistent with the possibility that low estrogens are driving *COMT* effects, as EE and endogenous estrogens are expected to be lowest in OC ~24 hours after pill ingestion. However, we are cautious to not overinterpret this pattern because pill time did not reliably modulate the influence of *COMT* (i.e., interact with *COMT*’s influence) on 2-back lure accuracy. Further, the one study to date that examined menstrual cycle and *COMT*-working memory relationships [22], showed that differences between *met/met* and *val/val* polymorphism were amplified in women who were in their low E2 phase. Relatedly, the *COMT Val*^*158*^*Met* polymorphism affects 2-back performance, with *met/met* making more correct responses than *val/val* in postmenopausal women and middle-aged men–both whom have low E2 levels–but has no effect in middle-aged premenopausal women (not stratified by cycle phase) who have higher E2 levels [40]. Together these findings suggest suppressed endogenous estrogens in OC, not level of EE, affects working memory by unmasking the influence of *COMT*.

In NOC, we did not find a direct effect between 2-back performance and *COMT*. We found that *met/met* NOC performed significantly better on 2-back lures in the high E2, late follicular phase, while *val/val* and *met/val* performance was unaffected by cycle phase. These results are contrary to previous research suggesting that *met/met* NOC have improved working memory in the low E2 early follicular phase [22]. In this respect, our findings in NOC suggest that the more dopamine and estradiol, the better is working memory performance. This finding is in contradistinction to the inverted-U dopamine response curve. One reason may be because our cohort was small, although similar in size to the previous study in which there were 13 *val/val* and 8 *met/met* [22]; the current study had 27 *val/val* and 7 *met/met*. It may also be due to ethnic differences of our cohort. Only half of our NOC participants self-identified as Caucasian. In a mixed sex cohort that controlled for sex there was evidence of a working memory advantage to carrying the *val* allele in a healthy Chinese population [41]. This might suggest that the “optimal dopamine” of the inverted-U is established for Caucasian women; *met/met* NOC in our study who are not Caucasian may be further from this optimal point and thus, benefit from higher E2. It is also possible that imperfect counterbalancing of estrogen condition could have contributed to these differences; participants were genotyped post-study completion, resulting in a higher proportion of *met/met* who completed their second session in the late follicular phase (Table 1). However, we did statistically control for session in our models to account for these practice effects.

### Cognitive outcomes beyond working memory

While there were no effects or interactions of *COMT* on AX-CPT, we found that OC had a significantly higher proactive behavioural index for reaction time than NOC in the AX-CPT. We interpret this finding as increased proactive control in OC. Proactive control refers to the maintenance of goal-relevant information to guide behavioural responses, and is mediated by frontal cortex [42]. Previous research examining inhibition of prepotent responses in women during different menstrual phases found that women in the follicular phase (high E2) were not as efficient at inhibiting prepotent responses on the stop-signal task as in their luteal or menstruation phase (low E2; [43]). In the same study, a significant positive correlation was observed between salivary E2 levels and mean stop signal reaction times, implying E2 is related to poorer inhibitory control [43]. This suggests higher E2 in our NOC participants may play a role in late-acting, stimulus driven, reactive control strategies, ultimately leading to longer reaction times on BX trials than AY trials, and hence, smaller proactive behavioural indices. With significantly lower circulating endogenous E2, OC proactive control may be promoted, driving more positive proactive behavioural indices in this group than in NOC.

More proactive control could also be explained by a pre-existing group difference. The OC group was recruited *because* they were taking OCs, a proactive measure against pregnancy. Thus, OC may have already been a group with higher proactive control in daily life decisions. Additionally, it is possible that OC may have had higher socio-economic status than NOC. Affordability is one of the main factors that affects a woman’s contraception use [44]. Higher socioeconomic status has been linked to a number of positive cognitive outcomes, including improved working memory, inhibition, and cognitive flexibility [45]. It would be interesting to compare women who use non-hormonal contraception, controlling for socioeconomic status, to determine if proactive control is related to EE or the personality type who takes OCs.

In OC, the short term memory test, Forward DS, performance was not directly affected by *COMT*, EE condition, or their interactions. However, in NOC, *met/met* showed better performance than *met/val* in the high E2 late follicular phase on the Forward DS. A mixed-sex study using the Stroop task found that the *met* allele was beneficial for cognitive stability of information [46]. However, rodent research suggests the inverted-U relationship between dopamine and working memory may be only observed under specific conditions of pharmacological enhancement or suppression of dopamine, and depends on the type of executive function being tested [47]. As with the n-back, our findings for NOC do not support the inverted-U dopamine response curve, and while it is consistent with our 2-back findings suggesting that enhanced dopamine may lead to better performance, it may be due to this task assessing information capacity rather than working memory, small sample size, counterbalancing, or ethnicity, as discussed earlier.

We did not find any effects or interactions between, *COMT*, estrogen condition, and group on the Backward DS or Digit Ordering task, two other tasks that require working memory. Like other young healthy participants, our participants likely devised grouping strategies to improve performance [48], ultimately leading to a ceiling effect.

### Strengths, limitations and future directions

This study considered endocrine and genetic status as mediators of the effects of OCs on working memory. While these have been underconsidered in memory studies, the effects of OCs in particular, have been understudied given that they are widely used over long terms.

This study is the first to consider the effect of the pharmacokinetic properties of OCs and genetics on working memory in women taking OCs. By studying the combination of these different factors on working memory, it demonstrates that the *COMT Val*^*158*^*Met* polymorphism plays a role in the working memory of OC, interacts with E2 in working memory and affects short term memory of NOC. These results highlight that genetic polymorphisms may interact with estrogens and should be considered when studying working and short term memory.

As with many genetic polymorphism studies, ours has the common limitation of a low number of participants carrying each polymorphism. Few women had the *met/met* polymorphism, reducing the power of our methods. However, the numbers in this study are within the range of others correlating neuropsychological measures with genetic polymorphisms [22,40,49,50]. Future research should consider using databases with available genetic data and correspondingly challenging neuropsychological tasks. The strength of our study is we administered two difficult working memory tasks not currently included in the extant large databases.

Another weakness is that we did not measure hormones directly. Our method of cycle phase determination was based on forward counting from menstruation onset, which is less reliable. However, NOC in our study were asked to reference period tracking applications or logs when booking sessions and completing questionnaires. This likely increased cycle phase determination beyond forward counting alone. Another weakness of our study, shared by most other studies of OC and cognition, is the variety of OCs formulations used. OCs can contain progestins of varying androgenicity; those with higher androgenicity have been correlated with better visuospatial ability [51]. Future studies on larger OC samples should group performance according to progestin type. Additionally, future research should investigate the pharmacokinetics of OCs and other hormonal contraceptives on other types of memory. Although there were no effects of pill ingestion time in the present study, this does not mean other cognitive domains may not be sensitive to daily EE fluctuations, or that the pharmacokinetics of other hormonal contraceptives with differing routes of administration (vaginal, transdermal, intramuscular, etc.) may not also influence cognition.

## Conclusions

Despite the widespread use of OCs, our knowledge about their impact on the brain and behaviour is limited. There has been no previous study of either the pharmacokinetics of EE on cognition, or its interactions with neurotransmitter systems, two important variables that could affect cognition. We investigated how the pharmacokinetics of EE in concert with the *COMT Val*^*158*^*Met* genetic polymorphism affected working memory comparing women taking OCs with those not taking them. Surprisingly, we found that the pharmacokinetics of EE did not affect either working or short-term memory. We did find, based on pill ingestion time, that it is likely suppressed endogenous E2–not EE levels–that affect working memory, including interacting with *COMT*. We also found that in our cohort, OCs contributed to proactive control, which may also be the result of lowered endogenous E2 in women taking OCs. These findings suggest that an important area for further investigation is the interaction between EE and endogenous estrogens in women who take OCs. As well, we hope this project inspires other researchers to investigate other aspects of cognition that may be influenced by the use of OCs.

## Supporting information

S1 File (DOCX)
