# Supplementary material for: Do oral contraceptives affect young women’s memory? Dopamine-dependent working memory is influenced by COMT genotype, but not time of pill ingestion
Source: PLoS One. 2021 Jun 10;16(6):e0252807. doi: 10.1371/journal.pone.0252807 (PMC8192013; doi:10.1371/journal.pone.0252807)
Supplement: S1 File — (DOCX) [file pone.0252807.s001.docx]

**S1 Table. Description of model variables.**

| Variable | Description | Type |
| --- | --- | --- |
| COMT | *COMT* genotype with 3 levels: “*met/met*”, “*met/val*”, “*val/val*” | Factor |
| COMT.effcode | *COMT* genotype effect coded: -1 = “*met/met*”, 0 = “*met/val*”, 1 = “*val/val*” | Numeric |
| estro_cond | Estrogen condition: -1 = low, 1 = high | Numeric |
| c.CESD | Grand mean centered CES-D score | Numeric |
| participant | Participant ID | Factor |
| session | Session: -1 = first session, 1 = second session | Numeric |
| blockcode | N-back type w/ 2 levels: “1_ntask”, “2_ntask” | Factor |
| log.RT | Log transformed reaction time | Numeric |
| correct | Accuracy; 1 = correct, 0 = incorrect | Integer |
| forward | Score on the forward digit span task | Integer |
| backward | Score on the backward digit span task | Integer |
| PBI_acc | Proactive behavioural index (PBI) for accuracy | Numeric |
| PBI_rt | Proactive behavioural index (PBI) for reaction time | Numeric |
| Total_errors | Sum of the number of repetitions and omissions | Integer |
| group | Participant group: -1 = women NOC, 1 = women OC | Numeric |
| trialcode | Trial type with 3 levels: “lure”, “target”, “nontarget” | Factor |

**S2 Table. Lure 2-back accuracy model output – COMT coded as a factor.**

glmer(correct ~ COMT*estro_cond + session + c.CESD + (estro_cond|participant), family=binomial, control=glmerControl(optimizer=“bobyqa”,optCtrl = list(maxfun = 100000)), subset = (blockcode==“2_ntask” & trialcode==“lure”), data = acc_df)

| Model term | Estimate | Std. Error | z value | Pr(>\|z\|) |
| --- | --- | --- | --- | --- |
| (Intercept) | 1.4211627 | 0.16369167 | 8.68194879 | 3.8904E-18 |
| met/val | -0.2578709 | 0.18618312 | -1.3850392 | 0.16604049 |
| val/val | -0.4691808 | 0.19669565 | -2.3853137 | 0.01706456 |
| estro_cond | 0.19488036 | 0.12189335 | 1.59877767 | 0.10987001 |
| group | 0.05827364 | 0.16327088 | 0.35691385 | 0.7211563 |
| session | 0.16896009 | 0.04582056 | 3.68742989 | 0.00022653 |
| c.CESD | 0.0089131 | 0.05878105 | 0.15163227 | 0.87947698 |
| met/val:estro_cond | -0.2696753 | 0.13610145 | -1.9814286 | 0.04754323 |
| val/val:estro_cond | -0.128516 | 0.14416582 | -0.8914456 | 0.37269015 |
| met/val:group | -0.0836474 | 0.18615607 | -0.4493402 | 0.65318629 |
| val/val:group | -0.1433781 | 0.19634077 | -0.7302514 | 0.46523655 |
| estro_cond:group | -0.3222779 | 0.11954562 | -2.6958569 | 0.00702078 |
| met/val:estro_cond:group | 0.3809441 | 0.13495561 | 2.82273633 | 0.00476157 |
| val/val:estro_cond:group | 0.41484378 | 0.14119579 | 2.93807465 | 0.00330257 |

**S3 Table. Lure 2-back accuracy reduced NOC model output – COMT coded as a factor.**

glmer(correct ~ COMT*estro_cond + session + c.CESD + (estro_cond|participant), family=binomial, control=glmerControl(optimizer=“bobyqa”,optCtrl = list(maxfun = 100000)), subset = (blockcode==“2_ntask” & trialcode==“lure” & group==-1), data = acc_df)

| Model term | Estimate | Std. Error | z value | Pr(>\|z\|) |
| --- | --- | --- | --- | --- |
| (Intercept) | 1.36276518 | 0.28096253 | 4.85034483 | 1.2325E-06 |
| met/val | -0.1698908 | 0.31234952 | -0.5439125 | 0.58650169 |
| val/val | -0.3434974 | 0.31326877 | -1.0964943 | 0.27286252 |
| estro_cond | 0.54725582 | 0.17263038 | 3.17010141 | 0.00152386 |
| session | 0.20122048 | 0.05423593 | 3.71009523 | 0.00020718 |
| c.CESD | 0.06151619 | 0.06820116 | 0.90198153 | 0.36706668 |
| met/val:estro_cond | -0.6545022 | 0.18784409 | -3.4842845 | 0.00049345 |
| val/val:estro_cond | -0.5493559 | 0.19017743 | -2.8886493 | 0.003869 |

**S4 Table. Lure 2-back accuracy reduced OC model output – COMT coded as a factor.**

glmer(correct ~ COMT*estro_cond + session + c.CESD + (estro_cond|participant), family=binomial, control=glmerControl(optimizer=“bobyqa”,optCtrl = list(maxfun = 100000)), subset = (blockcode==“2_ntask” & trialcode==“lure” & group==1), data = acc_df)

| Model term | Estimate | Std. Error | z value | Pr(>\|z\|) |
| --- | --- | --- | --- | --- |
| (Intercept) | 1.46693772 | 0.18210903 | 8.05527173 | 7.9302E-16 |
| met/val | -0.3386545 | 0.21466183 | -1.5776187 | 0.11465323 |
| val/val | -0.6043657 | 0.24365604 | -2.4804052 | 0.01312332 |
| estro_cond | -0.1711742 | 0.16560048 | -1.0336576 | 0.30129628 |
| session | 0.11198104 | 0.07538755 | 1.48540499 | 0.13743658 |
| c.CESD | -0.0620553 | 0.0994158 | -0.6241998 | 0.53249635 |
| met/val:estro_cond | 0.14554325 | 0.19108088 | 0.761684 | 0.44624863 |
| val/val:estro_cond | 0.31205461 | 0.2148555 | 1.45239291 | 0.14639239 |

**S5 Table. Lure 2-back reaction time model output – COMT coded as a factor.**

lmer(log.RT ~ COMT*estro_cond*group + session + c.CESD + (estro_cond|participant), control=lmerControl(optimizer=“bobyqa”,optCtrl = list(maxfun = 100000)), subset = (blockcode==“2_ntask” & trialcode==“lure”), data = rt_df)

| Model term | Estimate | Std. Error | df | t value | Pr(>\|t\|) |
| --- | --- | --- | --- | --- | --- |
| (Intercept) | 6.552723 | 0.038514 | 110.4077 | 170.1382 | 1.9E-135 |
| met/val | 0.034083 | 0.044256 | 110.5748 | 0.770128 | 0.442866 |
| val/val | -0.00415 | 0.047191 | 111.4318 | -0.08792 | 0.930096 |
| estro_cond | 0.01808 | 0.019026 | 104.2874 | 0.950284 | 0.344165 |
| group | -0.04548 | 0.038514 | 110.4013 | -1.1808 | 0.240221 |
| session | -0.04692 | 0.007772 | 108.9545 | -6.03752 | 2.21E-08 |
| c.CESD | -0.0008 | 0.001761 | 103.291 | -0.45418 | 0.650655 |
| met/val:estro_cond | -0.03326 | 0.021577 | 104.1803 | -1.54165 | 0.126191 |
| val/val:estro_cond | -0.00789 | 0.02366 | 107.1572 | -0.33353 | 0.739385 |
| met/val:group | 0.024476 | 0.044256 | 110.5761 | 0.55306 | 0.581339 |
| val/val:group | 0.054468 | 0.047191 | 111.4338 | 1.154201 | 0.250887 |
| estro_cond:group | -0.0141 | 0.018747 | 103.0299 | -0.75212 | 0.453693 |
| met/val:estro_cond:group | 0.01818 | 0.021321 | 103.5662 | 0.852674 | 0.395808 |
| val/val:estro_cond:group | 0.025812 | 0.022912 | 105.3633 | 1.126566 | 0.262486 |

**S6 Table. Target 2-back accuracy model output – COMT coded as a factor.**

glmer(correct ~ COMT*estro_cond*group + session + c.CESD + (estro_cond|participant), family=binomial, control=glmerControl(optimizer=“bobyqa”,optCtrl = list(maxfun = 100000)), subset = (blockcode==“2_ntask” & trialcode==“target”), data = acc_df)

| Model term | Estimate | Std. Error | z value | Pr(>\|z\|) | FDR correction |
| --- | --- | --- | --- | --- | --- |
| (Intercept) | 1.312101 | 0.163765 | 8.012087 | 1.13E-15 | 1.58E-14 |
| met/val | 0.077526 | 0.188289 | 0.411742 | 0.680528 | 0.866127 |
| val/val | -0.13982 | 0.200703 | -0.69665 | 0.486024 | 0.700598 |
| estro_cond | 0.09487 | 0.127823 | 0.742199 | 0.457967 | 0.700598 |
| group | 0.019701 | 0.163481 | 0.120508 | 0.904081 | 0.980871 |
| session | 0.198179 | 0.05169 | 3.833983 | 0.000126 | 0.001177 |
| c.CESD | 0.014219 | 0.007532 | 1.887837 | 0.059048 | 0.300607 |
| met/val:estro_cond | 0.046271 | 0.144871 | 0.319393 | 0.749428 | 0.87649 |
| val/val:estro_cond | 0.008071 | 0.15648 | 0.051579 | 0.958864 | 0.980871 |
| met/val:group | -0.0047 | 0.188256 | -0.02499 | 0.980065 | 0.980871 |
| val/val:group | 0.234524 | 0.200366 | 1.170478 | 0.241809 | 0.541651 |
| estro_cond:group | -0.09131 | 0.124853 | -0.73138 | 0.464548 | 0.700598 |
| met/val:estro_cond:group | 0.13661 | 0.143328 | 0.953132 | 0.340523 | 0.657836 |
| val/val:estro_cond:group | 0.232579 | 0.152496 | 1.525147 | 0.127222 | 0.445278 |

**S7 Table. Non-target 2-back accuracy model output – COMT coded as a factor.**

glmer(correct ~ COMT*estro_cond*group + session + c.CESD + (estro_cond|participant), family=binomial, control=glmerControl(optimizer=“bobyqa”,optCtrl = list(maxfun = 100000)), subset = (blockcode==“2_ntask” & trialcode==“nontarget”), data = acc_df)

| Model term | Estimate | Std. Error | z value | Pr(>\|z\|) | FDR  correction |
| --- | --- | --- | --- | --- | --- |
| (Intercept) | 4.258399 | 0.284954 | 14.94416 | 1.7E-50 | 9.52E-49 |
| met/val | -0.46442 | 0.316339 | -1.4681 | 0.142078 | 0.468021 |
| val/val | -0.613 | 0.33216 | -1.84549 | 0.064966 | 0.303173 |
| estro_cond | 0.35601 | 0.213804 | 1.665117 | 0.095889 | 0.383557 |
| group | -0.31682 | 0.282341 | -1.12211 | 0.261817 | 0.563913 |
| session | 0.515253 | 0.068914 | 7.476765 | 7.62E-14 | 8.53E-13 |
| c.CESD | 0.000272 | 0.011355 | 0.023976 | 0.980871 | 0.980871 |
| met/val:estro_cond | -0.27863 | 0.229743 | -1.21279 | 0.225208 | 0.525486 |
| val/val:estro_cond | -0.40217 | 0.238585 | -1.68564 | 0.091865 | 0.383557 |
| met/val:group | 0.396141 | 0.316924 | 1.249955 | 0.211316 | 0.514508 |
| val/val:group | 0.316476 | 0.332136 | 0.952852 | 0.340665 | 0.657836 |
| estro_cond:group | -0.44115 | 0.208054 | -2.12034 | 0.033977 | 0.190271 |
| met/val:estro_cond:group | 0.585441 | 0.228446 | 2.562704 | 0.010386 | 0.072702 |
| val/val:estro_cond:group | 0.565371 | 0.236425 | 2.391336 | 0.016787 | 0.104454 |

**S8 Table. Target 1-back accuracy model output – COMT coded as a factor.**

glmer(correct ~ COMT*estro_cond*group + session + c.CESD + (estro_cond|participant), family=binomial, control=glmerControl(optimizer=“bobyqa”,optCtrl = list(maxfun = 100000)), subset = (blockcode==“1_ntask” & trialcode==”target”), data = acc_df)

| Model term | Estimate | Std. Error | z value | Pr(>\|z\|) | FDR correction |
| --- | --- | --- | --- | --- | --- |
| (Intercept) | 2.03286 | 0.234111 | 8.683326 | 3.84E-18 | 7.17E-17 |
| met/val | 0.192403 | 0.268141 | 0.717546 | 0.473037 | 0.700598 |
| val/val | -0.01748 | 0.282747 | -0.06181 | 0.950713 | 0.980871 |
| estro_cond | -0.23674 | 0.168531 | -1.4047 | 0.16011 | 0.471903 |
| group | 0.17121 | 0.23149 | 0.739601 | 0.459542 | 0.700598 |
| session | -0.05602 | 0.067232 | -0.83316 | 0.404754 | 0.700598 |
| c.CESD | -0.00333 | 0.010355 | -0.32173 | 0.747657 | 0.87649 |
| met/val:estro_cond | 0.289624 | 0.18876 | 1.534344 | 0.124945 | 0.445278 |
| val/val:estro_cond | 0.250617 | 0.199412 | 1.256782 | 0.208833 | 0.514508 |
| met/val:group | 0.066385 | 0.267871 | 0.247826 | 0.804269 | 0.919164 |
| val/val:group | -0.37168 | 0.282659 | -1.31494 | 0.188529 | 0.514508 |
| estro_cond:group | 0.146011 | 0.159873 | 0.913295 | 0.361087 | 0.67403 |
| met/val:estro_cond:group | -0.06275 | 0.18506 | -0.33906 | 0.734567 | 0.87649 |
| val/val:estro_cond:group | -0.08251 | 0.194214 | -0.42484 | 0.670955 | 0.866127 |

**S9 Table. Non-target 1-back accuracy model output – COMT coded as a factor.**

glmer(correct ~ COMT*estro_cond*group + session + c.CESD + (estro_cond|participant), family=binomial, control=glmerControl(optimizer=“bobyqa”,optCtrl = list(maxfun = 100000)), subset = (blockcode==“1_ntask” & trialcode==“nontarget”), data = acc_df)

| Model term | Estimate | Std. Error | z value | Pr(>\|z\|) | FDR correction |
| --- | --- | --- | --- | --- | --- |
| (Intercept) | 5.355433 | 0.395116 | 13.55409 | 7.49E-42 | 2.1E-40 |
| met/val | -0.53555 | 0.42758 | -1.25252 | 0.21038 | 0.514508 |
| val/val | -0.63837 | 0.449259 | -1.42094 | 0.155334 | 0.471903 |
| estro_cond | -0.27342 | 0.373701 | -0.73166 | 0.464377 | 0.700598 |
| group | -0.26561 | 0.382931 | -0.69363 | 0.487916 | 0.700598 |
| session | 0.417173 | 0.123903 | 3.366928 | 0.00076 | 0.006081 |
| c.CESD | -0.00978 | 0.01663 | -0.58837 | 0.556281 | 0.778794 |
| met/val:estro_cond | 0.324683 | 0.399202 | 0.81333 | 0.416029 | 0.700598 |
| val/val:estro_cond | 0.073703 | 0.420046 | 0.175463 | 0.860716 | 0.964002 |
| met/val:group | 0.135527 | 0.427592 | 0.316955 | 0.751277 | 0.87649 |
| val/val:group | 0.438318 | 0.449751 | 0.97458 | 0.329769 | 0.657836 |
| estro_cond:group | 0.159805 | 0.359706 | 0.444266 | 0.65685 | 0.866127 |
| met/val:estro_cond:group | -0.21725 | 0.399994 | -0.54312 | 0.587046 | 0.801819 |
| val/val:estro_cond:group | -0.0464 | 0.415935 | -0.11156 | 0.911172 | 0.980871 |

**S10 Table. Target 2-back reaction time model output – COMT coded as a factor.**

lmer(log.RT ~ COMT*estro_cond*group + session + c.CESD + (estro_cond|participant), control=lmerControl(optimizer=”bobyqa”,optCtrl = list(maxfun = 100000)), subset = (blockcode==“2_ntask” & trialcode==”target “), data = rt_df)

| Model term | Estimate | Std. Error | df | t value | Pr(>\|t\|) | FDR correction |
| --- | --- | --- | --- | --- | --- | --- |
| (Intercept) | 6.430486 | 0.036818 | 111.8032 | 174.6566 | 4.1E-138 | 5.8E-137 |
| met/val | 0.031019 | 0.042285 | 111.7406 | 0.733575 | 0.464744 | 0.765461 |
| val/val | 0.012947 | 0.045055 | 112.3452 | 0.287353 | 0.774371 | 0.903797 |
| estro_cond | 0.014635 | 0.014709 | 105.6212 | 0.995024 | 0.321999 | 0.693536 |
| group | -0.04556 | 0.036818 | 111.8034 | -1.23744 | 0.218516 | 0.506457 |
| session | -0.03666 | 0.005928 | 109.3926 | -6.18379 | 1.1E-08 | 7.73E-08 |
| c.CESD | -0.00173 | 0.001332 | 102.2507 | -1.29912 | 0.196824 | 0.497975 |
| met/val:estro_cond | -0.00623 | 0.016633 | 104.1951 | -0.37441 | 0.708862 | 0.899679 |
| val/val:estro_cond | -0.01062 | 0.018249 | 106.7751 | -0.5822 | 0.56166 | 0.827709 |
| met/val:group | 0.018087 | 0.042284 | 111.732 | 0.427749 | 0.669657 | 0.892876 |
| val/val:group | 0.057742 | 0.045055 | 112.3422 | 1.281602 | 0.202621 | 0.497975 |
| estro_cond:group | -0.02049 | 0.01451 | 104.5355 | -1.41228 | 0.160839 | 0.485266 |
| met/val:estro_cond:group | 0.013127 | 0.016511 | 105.1088 | 0.795056 | 0.428372 | 0.741299 |
| val/val:estro_cond:group | 0.027434 | 0.017717 | 105.5627 | 1.548473 | 0.124501 | 0.459154 |

**S11 Table. Non-target 2-back reaction time model output – *COMT* coded as a factor.**

lmer(log.RT ~ COMT*estro_cond*group + session + c.CESD + (estro_cond|participant), control=lmerControl(optimizer=”bobyqa”,optCtrl = list(maxfun = 100000)), subset = (blockcode==“2_ntask” & trialcode==”nontarget “), data = rt_df)

| Model term | Estimate | Std. Error | df | t value | Pr(>\|t\|) | FDR correction |
| --- | --- | --- | --- | --- | --- | --- |
| (Intercept) | 6.19624 | 0.031338 | 111.3884 | 197.7234 | 1.1E-143 | 1.4E-138 |
| met/val | 0.001022 | 0.036011 | 111.5425 | 0.028377 | 0.977412 | 0.971706 |
| val/val | -0.01864 | 0.0383 | 111.4153 | -0.48665 | 0.62746 | 0.892876 |
| estro_cond | -0.02045 | 0.014617 | 108.3727 | -1.39908 | 0.164644 | 0.903797 |
| group | -0.05554 | 0.031338 | 111.3885 | -1.77231 | 0.079077 | 0.703463 |
| session | -0.04569 | 0.005871 | 108.9844 | -7.78269 | 4.36E-12 | 6.05E-12 |
| c.CESD | -0.00058 | 0.001351 | 108.4944 | -0.43028 | 0.667848 | 0.98303 |
| met/val:estro_cond | 0.038944 | 0.016599 | 108.3226 | 2.34624 | 0.020783 | 0.901123 |
| val/val:estro_cond | 0.015011 | 0.01793 | 108.5977 | 0.837169 | 0.404336 | 0.98303 |
| met/val:group | 0.045959 | 0.036011 | 111.5425 | 1.276229 | 0.204526 | 0.903797 |
| val/val:group | 0.013612 | 0.0383 | 111.4152 | 0.355407 | 0.722957 | 0.703463 |
| estro_cond:group | -0.01147 | 0.014452 | 108.2884 | -0.79385 | 0.42902 | 0.459154 |
| met/val:estro_cond:group | 0.005874 | 0.016433 | 108.2861 | 0.357445 | 0.721455 | 0.485266 |
| val/val:estro_cond:group | 0.010874 | 0.017467 | 108.3279 | 0.622542 | 0.534894 | 0.284569 |

**S12 Table. Target 1-back reaction time model output – *COMT* coded as a factor.**

lmer(log.RT ~ COMT*estro_cond*group + session + c.CESD + (estro_cond|participant), control=lmerControl(optimizer=”bobyqa”,optCtrl = list(maxfun = 100000)), subset = (blockcode==“1_ntask” & trialcode==”target “), data = rt_df)

| Model term | Estimate | Std. Error | df | t value | Pr(>\|t\|) | FDR correction |
| --- | --- | --- | --- | --- | --- | --- |
| (Intercept) | 6.216081 | 0.030956 | 113.4101 | 200.8056 | 1.4E-146 | 8E-145 |
| met/val | 0.021698 | 0.035529 | 113.0635 | 0.61072 | 0.542611 | 0.821249 |
| val/val | -0.02951 | 0.037819 | 113.2915 | -0.78031 | 0.436837 | 0.741299 |
| estro_cond | -0.02395 | 0.013743 | 108.5755 | -1.74245 | 0.084261 | 0.362972 |
| group | -0.04992 | 0.030956 | 113.4116 | -1.61253 | 0.109625 | 0.438501 |
| session | -0.03483 | 0.005473 | 105.8053 | -6.36446 | 5.13E-09 | 4.11E-08 |
| c.CESD | 0.000292 | 0.00125 | 104.2142 | 0.233829 | 0.815576 | 0.913445 |
| met/val:estro_cond | 0.030012 | 0.01554 | 107.5676 | 1.931277 | 0.05608 | 0.2855 |
| val/val:estro_cond | 0.021596 | 0.016864 | 109.3734 | 1.280595 | 0.203045 | 0.497975 |
| met/val:group | 0.043243 | 0.035529 | 113.0637 | 1.217109 | 0.226097 | 0.506457 |
| val/val:group | 0.002569 | 0.037819 | 113.292 | 0.067934 | 0.945958 | 0.98303 |
| estro_cond:group | -0.00931 | 0.013621 | 109.3743 | -0.68345 | 0.495771 | 0.793233 |
| met/val:estro_cond:group | 0.013851 | 0.015437 | 108.5179 | 0.897223 | 0.371586 | 0.717546 |
| val/val:estro_cond:group | 0.003468 | 0.016443 | 109.3963 | 0.210881 | 0.833373 | 0.915076 |

**S13 Table. Non-target 1-back reaction time model output – *COMT* coded as a factor.**

lmer(log.RT ~ COMT*estro_cond*group + session + c.CESD + (estro_cond|participant), control=lmerControl(optimizer=”bobyqa”,optCtrl = list(maxfun = 100000)), subset = (blockcode==“1_ntask” & trialcode==”nontarget”), data = rt_df)

| Model term | Estimate | Std. Error | df | t value | Pr(>\|t\|) | FDR correction |
| --- | --- | --- | --- | --- | --- | --- |
| (Intercept) | 6.19624 | 0.031338 | 111.3884 | 197.7234 | 1.1E-143 | 3.2E-142 |
| met/val | 0.001022 | 0.036011 | 111.5425 | 0.028377 | 0.977412 | 0.98303 |
| val/val | -0.01864 | 0.0383 | 111.4153 | -0.48665 | 0.62746 | 0.892876 |
| estro_cond | -0.02045 | 0.014617 | 108.3727 | -1.39908 | 0.164644 | 0.485266 |
| group | -0.05554 | 0.031338 | 111.3885 | -1.77231 | 0.079077 | 0.362972 |
| session | -0.04569 | 0.005871 | 108.9844 | -7.78269 | 4.36E-12 | 4.07E-11 |
| c.CESD | -0.00058 | 0.001351 | 108.4944 | -0.43028 | 0.667848 | 0.892876 |
| met/val:estro_cond | 0.038944 | 0.016599 | 108.3226 | 2.34624 | 0.020783 | 0.129316 |
| val/val:estro_cond | 0.015011 | 0.01793 | 108.5977 | 0.837169 | 0.404336 | 0.741299 |
| met/val:group | 0.045959 | 0.036011 | 111.5425 | 1.276229 | 0.204526 | 0.497975 |
| val/val:group | 0.013612 | 0.0383 | 111.4152 | 0.355407 | 0.722957 | 0.899679 |
| estro_cond:group | -0.01147 | 0.014452 | 108.2884 | -0.79385 | 0.42902 | 0.741299 |
| met/val:estro_cond:group | 0.005874 | 0.016433 | 108.2861 | 0.357445 | 0.721455 | 0.899679 |
| val/val:estro_cond:group | 0.010874 | 0.017467 | 108.3279 | 0.622542 | 0.534894 | 0.821249 |

**S14 Table. Lure 2-back accuracy model output – *COMT* coded as a numeric.**

glmer(correct ~ COMT.effcode*estro_cond*group + session + c.CESD + (estro_cond|participant), family=binomial, control=glmerControl(optimizer=”bobyqa”,optCtrl = list(maxfun = 100000)), subset = (blockcode==“2_ntask” & trialcode==“lure”), data = acc_df)

| Model terms | Estimate | Std. Error | z value | Pr(>\|z\|) |
| --- | --- | --- | --- | --- |
| (Intercept) | 1.16972983 | 0.06650116 | 17.5896161 | 2.9588E-69 |
| COMT.effcode | -0.2233641 | 0.09206229 | -2.4262277 | 0.01525669 |
| estro_cond | 0.01409973 | 0.04979367 | 0.28316317 | 0.77705176 |
| group | -0.0135875 | 0.06567511 | -0.2068904 | 0.83609544 |
| session | 0.17122153 | 0.04674386 | 3.66297352 | 0.0002493 |
| c.CESD | 1.1485E-05 | 0.00711864 | 0.00161341 | 0.99871269 |
| COMT.effcode:estro_cond | 0.00159506 | 0.06886682 | 0.02316152 | 0.98152143 |
| COMT.effcode:group | -0.0821676 | 0.09171337 | -0.8959176 | 0.37029679 |
| estro_cond:group | -0.0143831 | 0.04862639 | -0.295788 | 0.76739197 |
| COMT.effcode:estro_cond:group | 0.14110678 | 0.0671295 | 2.10200858 | 0.03555252 |

**S15 Table. Lure 2-back accuracy reduced OC model output – *COMT* coded as a numeric.**

glmer(correct ~ COMT.effcode*estro_cond + session + c.CESD + (estro_cond|participant), family=binomial, control=glmerControl(optimizer=”bobyqa”,optCtrl = list(maxfun = 100000)), subset = (blockcode==“2_ntask” & trialcode==”lure” & group==1), data = acc_df)

| Model term | Estimate | Std. Error | z value | Pr(>\|z\|) |
| --- | --- | --- | --- | --- |
| (Intercept) | 1.14565457 | 0.08561355 | 13.3816969 | 7.7361E-41 |
| COMT.effcode | -0.3005616 | 0.12139323 | -2.4759338 | 0.01328882 |
| estro_cond | -0.0206598 | 0.07443851 | -0.2775415 | 0.78136432 |
| session | 0.11068074 | 0.07439126 | 1.48781909 | 0.13679861 |
| c.CESD | -0.0074889 | 0.01208004 | -0.6199413 | 0.53529642 |
| COMT.effcode:estro_cond | 0.15663706 | 0.10654404 | 1.47016251 | 0.14151775 |

**S16 Table. Lure 2-back accuracy reduced NOC model output – *COMT* coded as a numeric.**

glmer(correct ~ COMT.effcode*estro_cond + session + c.CESD + (estro_cond|participant), family=binomial, control=glmerControl(optimizer=”bobyqa”,optCtrl = list(maxfun = 100000)), subset = (blockcode==“2_ntask” & trialcode==“lure” & group==-1), data = acc_df)

| Model term | Estimate | Std. Error | z value | Pr(>\|z\|) |
| --- | --- | --- | --- | --- |
| (Intercept) | 1.18444585 | 0.10268329 | 11.534942 | 8.7946E-31 |
| COMT.effcode | -0.151377 | 0.1384431 | -1.0934238 | 0.27420779 |
| estro_cond | 0.05123142 | 0.06699118 | 0.76474873 | 0.44442118 |
| session | 0.21082883 | 0.05773121 | 3.65190406 | 0.0002603 |
| c.CESD | 0.00458513 | 0.00839638 | 0.54608446 | 0.58500788 |
| COMT.effcode:estro_cond | -0.1464486 | 0.08823588 | -1.6597397 | 0.09696683 |

**S17 Table. Lure 2-back reaction time model output – *COMT* coded as a numeric.**

lmer(log.RT ~ COMT.effcode*estro_cond + session + c.CESD + (estro_cond|participant), control=lmerControl(optimizer=“bobyqa”,optCtrl = list(maxfun = 100000)), subset = (blockcode==“2_ntask” & trialcode==“lure“), data = rt_df)

| Model term | Estimate | Std. Error | df | t value | Pr(>\|t\|) |
| --- | --- | --- | --- | --- | --- |
| (Intercept) | 6.570507 | 0.015948 | 113.2333 | 411.9994 | 1.1E-181 |
| COMT.effcode | -0.00857 | 0.022255 | 113.7516 | -0.38487 | 0.70105 |
| estro_cond | -0.00211 | 0.007929 | 109.026 | -0.26586 | 0.790853 |
| group | -0.02058 | 0.015947 | 113.1959 | -1.29042 | 0.199533 |
| session | -0.04762 | 0.007798 | 111.4673 | -6.1071 | 1.52E-08 |
| c.CESD | -0.00105 | 0.001775 | 105.5349 | -0.59106 | 0.555741 |
| COMT.effcode:estro_cond | 0.001693 | 0.01141 | 110.9521 | 0.14834 | 0.882344 |
| COMT.effcode:group | 0.033894 | 0.022256 | 113.7556 | 1.52296 | 0.130544 |
| estro_cond:group | 0.002415 | 0.008053 | 108.7219 | 0.299876 | 0.764844 |
| COMT.effcode:estro_cond:group | 0.007034 | 0.010959 | 108.7183 | 0.641846 | 0.522324 |

**S18 Table. Target 2-back accuracy model output – *COMT* coded as a numeric.**

glmer(correct ~ COMT.effcode*estro_cond*group + session + c.CESD + (estro_cond|participant), family=binomial, control=glmerControl(optimizer=“bobyqa”,optCtrl = list(maxfun = 100000)), subset = (blockcode==“2_ntask” & trialcode==“target”), data = acc_df)

| Model term | Estimate | Std. Error | z value | Pr(>\|z\|) | FDR correction |
| --- | --- | --- | --- | --- | --- |
| (Intercept) | 1.323528 | 0.069968 | 18.91608 | 8.41E-80 | 8.41E-79 |
| COMT.effcode | -0.11253 | 0.096185 | -1.16992 | 0.242033 | 0.561855 |
| estro_cond | 0.121409 | 0.054078 | 2.245068 | 0.024764 | 0.123819 |
| group | 0.068175 | 0.068845 | 0.990275 | 0.32204 | 0.592324 |
| session | 0.199329 | 0.051361 | 3.880917 | 0.000104 | 0.000694 |
| c.CESD | 0.013008 | 0.007482 | 1.738678 | 0.082092 | 0.322145 |
| COMT.effcode:estro_cond | -0.0006 | 0.074355 | -0.00807 | 0.993565 | 0.993565 |
| COMT.effcode:group | 0.162409 | 0.095838 | 1.694634 | 0.090145 | 0.322145 |
| estro_cond:group | 0.036333 | 0.052564 | 0.691213 | 0.489432 | 0.783091 |
| COMT.effcode:estro_cond:group | 0.11981 | 0.072116 | 1.661349 | 0.096643 | 0.322145 |

**S19 Table. Non-target 2-back accuracy model output – *COMT* coded as a numeric.**

glmer(correct ~ COMT.effcode*estro_cond*group + session + c.CESD + (estro_cond|participant), family=binomial, control=glmerControl(optimizer=“bobyqa”,optCtrl = list(maxfun = 100000)), subset = (blockcode==“2_ntask” & trialcode==“nontarget”), data = acc_df)

| Model term | Estimate | Std. Error | z value | Pr(>\|z\|) | FDR correction |
| --- | --- | --- | --- | --- | --- |
| (Intercept) | 3.841681 | 0.114076 | 33.67657 | 1.27E-248 | 5.1E-247 |
| COMT.effcode | -0.20423 | 0.14898 | -1.37087 | 0.170416796 | 0.454445 |
| estro_cond | 0.082982 | 0.0835 | 0.993793 | 0.320323802 | 0.592324 |
| group | -0.01076 | 0.106638 | -0.10095 | 0.919593839 | 0.993565 |
| session | 0.519231 | 0.069622 | 7.457839 | 8.79531E-14 | 7.04E-13 |
| c.CESD | 0.000402 | 0.011355 | 0.035432 | 0.971735606 | 0.993565 |
| COMT.effcode:estro_cond | -0.11061 | 0.1021 | -1.08335 | 0.278652538 | 0.586637 |
| COMT.effcode:group | 0.070181 | 0.148592 | 0.472308 | 0.636706877 | 0.894276 |
| estro_cond:group | 0.025129 | 0.073654 | 0.34118 | 0.732968453 | 0.894276 |
| COMT.effcode:estro_cond:group | 0.18125 | 0.101001 | 1.794528 | 0.072728883 | 0.322145 |

**S20 Table. Target 1-back accuracy model output – *COMT* coded as a numeric.**

glmer(correct ~ COMT.effcode*estro_cond*group + session + c.CESD + (estro_cond|participant), family=binomial, control=glmerControl(optimizer=“bobyqa”,optCtrl = list(maxfun = 100000)), subset = (blockcode==“1_ntask” & trialcode==“target”), data = acc_df)

| Model term | Estimate | Std. Error | z value | Pr(>\|z\|) | FDR  correction |
| --- | --- | --- | --- | --- | --- |
| (Intercept) | 2.123688 | 0.103718 | 20.4756 | 3.55E-93 | 4.74E-92 |
| COMT.effcode | -0.0059 | 0.136995 | -0.0431 | 0.965621 | 0.993565 |
| estro_cond | -0.02364 | 0.077425 | -0.3053 | 0.760135 | 0.894276 |
| group | 0.112164 | 0.098089 | 1.143491 | 0.252835 | 0.561855 |
| session | -0.04819 | 0.066955 | -0.71979 | 0.471657 | 0.783091 |
| c.CESD | -0.00332 | 0.010352 | -0.32067 | 0.748463 | 0.894276 |
| COMT.effcode:estro_cond | 0.092012 | 0.096201 | 0.956459 | 0.33884 | 0.592324 |
| COMT.effcode:group | -0.2086 | 0.13684 | -1.52444 | 0.127399 | 0.391997 |
| estro_cond:group | 0.084966 | 0.068246 | 1.245001 | 0.213131 | 0.532829 |
| COMT.effcode:estro_cond:group | -0.01881 | 0.093727 | -0.20067 | 0.840955 | 0.961092 |

**S21 Table. Non-target 1-back accuracy model output – *COMT* coded as a numeric.**

glmer(correct ~ COMT.effcode*estro_cond*group + session + c.CESD + (estro_cond|participant), family=binomial, control=glmerControl(optimizer=“bobyqa”,optCtrl = list(maxfun = 100000)), subset = (blockcode==“1_ntask” & trialcode==“nontarget”), data = acc_df)

| Model term | Estimate | Std. Error | z value | Pr(>\|z\|) | FDR  correction |
| --- | --- | --- | --- | --- | --- |
| (Intercept) | 4.919684 | 0.172391 | 28.53796 | 4E-179 | 7.9E-178 |
| COMT.effcode | -0.27986 | 0.201131 | -1.39142 | 0.164099 | 0.454445 |
| estro_cond | -0.06834 | 0.163143 | -0.41891 | 0.675284 | 0.894276 |
| group | -0.07911 | 0.14417 | -0.5487 | 0.583212 | 0.894276 |
| session | 0.416605 | 0.124247 | 3.353027 | 0.000799 | 0.004568 |
| c.CESD | -0.00859 | 0.016617 | -0.51696 | 0.605182 | 0.894276 |
| COMT.effcode:estro_cond | -0.07319 | 0.187618 | -0.39013 | 0.696442 | 0.894276 |
| COMT.effcode:group | 0.191443 | 0.200883 | 0.953007 | 0.340586 | 0.592324 |
| estro_cond:group | 0.014998 | 0.13363 | 0.112235 | 0.910637 | 0.993565 |
| COMT.effcode:estro_cond:group | 0.075603 | 0.183886 | 0.411142 | 0.680969 | 0.894276 |

**S22 Table. Target 2-back reaction time model output – *COMT* coded as a numeric.**

lmer(log.RT ~ COMT.effcode*estro_cond + session + c.CESD + (estro_cond|participant), control=lmerControl(optimizer=“bobyqa”,optCtrl = list(maxfun = 100000)), subset = (blockcode==“2_ntask” & trialcode==“target“), data = rt_df)

| Model term | Estimate | Std. Error | df | t value | Pr(>\|t\|) | FDR correction |
| --- | --- | --- | --- | --- | --- | --- |
| (Intercept) | 6.450385 | 0.015185 | 113.9162 | 424.7908 | 4E-184 | 4E-183 |
| COMT.effcode | 0.000601 | 0.021195 | 114.6078 | 0.028361 | 0.977424 | 0.986704 |
| estro_cond | 0.008842 | 0.006013 | 108.9914 | 1.470604 | 0.144279 | 0.384745 |
| group | -0.02299 | 0.015185 | 113.9074 | -1.51381 | 0.132844 | 0.379554 |
| session | -0.0367 | 0.005826 | 110.5047 | -6.2986 | 6.27E-09 | 3.58E-08 |
| c.CESD | -0.00173 | 0.001318 | 104.2102 | -1.31021 | 0.193007 | 0.482517 |
| COMT.effcode:estro_cond | -0.00519 | 0.008659 | 111.0188 | -0.59947 | 0.55008 | 0.846277 |
| COMT.effcode:group | 0.034499 | 0.021195 | 114.61 | 1.627689 | 0.106337 | 0.327191 |
| estro_cond:group | -0.00708 | 0.006076 | 106.8074 | -1.16454 | 0.246801 | 0.493601 |
| COMT.effcode:estro_cond:group | 0.013605 | 0.008334 | 109.1515 | 1.632588 | 0.105437 | 0.327191 |

**S23 Table. Non-target 2-back reaction time model output – *COMT* coded as a numeric.**

lmer(log.RT ~ COMT.effcode*estro_cond + session + c.CESD + (estro_cond|participant), control=lmerControl(optimizer=“bobyqa”,optCtrl = list(maxfun = 100000)), subset = (blockcode==“2_ntask” & trialcode==“nontarget“), data = rt_df)

| Model term | Estimate | Std. Error | df | t value | Pr(>\|t\|) | FDR correction |
| --- | --- | --- | --- | --- | --- | --- |
| (Intercept) | 6.430081 | 0.01466 | 114.0226 | 438.6013 | 7.5E-186 | 1E-184 |
| COMT.effcode | -0.0139 | 0.02043 | 114.015 | -0.68048 | 0.49758 | 0.804279 |
| estro_cond | 0.001031 | 0.006196 | 112.2238 | 0.166362 | 0.868172 | 0.964635 |
| group | -0.01833 | 0.01466 | 114.0229 | -1.25023 | 0.213775 | 0.493601 |
| session | -0.05006 | 0.006054 | 112.7815 | -8.26875 | 2.98E-13 | 2.39E-12 |
| c.CESD | -7.6E-05 | 0.001397 | 112.0105 | -0.05455 | 0.956595 | 0.986704 |
| COMT.effcode:estro_cond | 0.002256 | 0.008817 | 112.4707 | 0.255862 | 0.798525 | 0.956009 |
| COMT.effcode:group | 0.024317 | 0.02043 | 114.0146 | 1.190235 | 0.236426 | 0.493601 |
| estro_cond:group | -0.00135 | 0.006302 | 112.3257 | -0.21355 | 0.831285 | 0.956009 |
| COMT.effcode:estro_cond:group | 0.015897 | 0.00853 | 112.1926 | 1.863716 | 0.064976 | 0.236277 |

**S24 Table. Target 1-back reaction time model output – *COMT* coded as a numeric.**

lmer(log.RT ~ COMT.effcode*estro_cond + session + c.CESD + (estro_cond|participant), control=lmerControl(optimizer=“bobyqa”,optCtrl = list(maxfun = 100000)), subset = (blockcode==“1_ntask” & trialcode==“target“), data = rt_df)

| Model term | Estimate | Std. Error | df | t value | Pr(>\|t\|) | FDR correction |
| --- | --- | --- | --- | --- | --- | --- |
| (Intercept) | 6.220187 | 0.012975 | 114.4079 | 479.3862 | 8.3E-191 | 3.3E-189 |
| COMT.effcode | -0.01462 | 0.018094 | 114.7254 | -0.80802 | 0.420749 | 0.731737 |
| estro_cond | -0.00267 | 0.005668 | 108.2305 | -0.47127 | 0.6384 | 0.945778 |
| group | -0.02674 | 0.012976 | 114.4268 | -2.0611 | 0.041559 | 0.166238 |
| session | -0.03359 | 0.0055 | 106.7641 | -6.10827 | 1.66E-08 | 8.32E-08 |
| c.CESD | 0.000397 | 0.001265 | 105.7935 | 0.313748 | 0.75433 | 0.956009 |
| COMT.effcode:estro_cond | 0.009004 | 0.008089 | 110.1018 | 1.113094 | 0.268092 | 0.510652 |
| COMT.effcode:group | -0.0003 | 0.018094 | 114.7229 | -0.0167 | 0.986704 | 0.986704 |
| estro_cond:group | -0.0014 | 0.005769 | 108.9725 | -0.24247 | 0.808873 | 0.956009 |
| COMT.effcode:estro_cond:group | 0.002914 | 0.00782 | 109.5652 | 0.372597 | 0.710168 | 0.956009 |

**S25 Table. Non-target 1-back reaction time model output – *COMT* coded as a numeric.** lmer(log.RT ~ COMT.effcode*estro_cond + session + c.CESD + (estro_cond|participant), control=lmerControl(optimizer=“bobyqa”,optCtrl = list(maxfun = 100000)), subset = (blockcode==“1_ntask” & trialcode==“nontarget“), data = rt_df)

| Model term | Estimate | Std. Error | df | t value | Pr(>\|t\|) | FDR correction |
| --- | --- | --- | --- | --- | --- | --- |
| (Intercept) | 6.191789 | 0.013041 | 113.4923 | 474.8031 | 5.1E-189 | 1E-187 |
| COMT.effcode | -0.00508 | 0.018159 | 113.2051 | -0.27972 | 0.780206 | 0.956009 |
| estro_cond | 0.004135 | 0.006149 | 110.6341 | 0.672489 | 0.502674 | 0.804279 |
| group | -0.02783 | 0.013041 | 113.4898 | -2.13411 | 0.034986 | 0.155494 |
| session | -0.04455 | 0.005975 | 110.9329 | -7.45658 | 2.1E-11 | 1.4E-10 |
| c.CESD | -0.00037 | 0.001381 | 110.3468 | -0.26859 | 0.788745 | 0.956009 |
| COMT.effcode:estro_cond | 0.001803 | 0.008717 | 110.7041 | 0.206849 | 0.836507 | 0.956009 |
| COMT.effcode:group | 0.001606 | 0.018159 | 113.205 | 0.088421 | 0.929698 | 0.986704 |
| estro_cond:group | -0.00667 | 0.006268 | 110.8176 | -1.06393 | 0.289675 | 0.526681 |
| COMT.effcode:estro_cond:group | 0.010348 | 0.008434 | 110.3969 | 1.226994 | 0.222435 | 0.493601 |

**S26 Table. Forward digit span model output – *COMT* coded as a factor.**

lmer(forward ~ group*COMT*estro_cond + (1|participant) + session + c.CESD, data = dataset)

| Model term | Estimate | Std. Error | df | t value | Pr(>\|t\|) |
| --- | --- | --- | --- | --- | --- |
| (Intercept) | 6.769712 | 0.227789 | 112.7609 | 29.71925 | 3.62E-55 |
| met/val | -0.0295 | 0.261649 | 112.7607 | -0.11276 | 0.910418 |
| val/val | -0.24924 | 0.277328 | 113.5823 | -0.89871 | 0.370707 |
| estro_cond | 0.202963 | 0.127474 | 111.7551 | 1.592191 | 0.114166 |
| group | -0.15849 | 0.227768 | 112.7394 | -0.69582 | 0.487974 |
| c.CESD | -0.00343 | 0.008484 | 223.6235 | -0.40382 | 0.686734 |
| session | 0.113779 | 0.051101 | 112.0138 | 2.226551 | 0.027978 |
| met/val:estro_cond | -0.32494 | 0.144645 | 111.6639 | -2.24644 | 0.026643 |
| val/val:estro_cond | -0.2055 | 0.155634 | 111.7664 | -1.32043 | 0.189391 |
| met/val:group | 0.189614 | 0.261629 | 112.7422 | 0.724745 | 0.47011 |
| val/val:group | -0.08933 | 0.276271 | 112.6914 | -0.32336 | 0.747025 |
| estro_cond:group | -0.19585 | 0.125239 | 113.1891 | -1.56377 | 0.120662 |
| met/val:estro_cond:group | 0.169227 | 0.143061 | 111.8391 | 1.182906 | 0.239355 |
| val/val:estro_cond:group | 0.255525 | 0.151007 | 112.1814 | 1.692137 | 0.093396 |

**S27 Table. Forward digit span reduced NOC model output – *COMT* coded as a factor.**

lmer(forward ~ COMT*estro_cond + (1|participant) + session + c.CESD, subset = (group==-1), data = dataset)

| Model term | Estimate | Std. Error | df | t value | Pr(>\|t\|) |
| --- | --- | --- | --- | --- | --- |
| (Intercept) | 6.927756 | 0.382698 | 58.80029 | 18.10241 | 1.02E-25 |
| met/val | -0.22482 | 0.428149 | 58.88846 | -0.5251 | 0.601484 |
| val/val | -0.1488 | 0.430512 | 59.13325 | -0.34564 | 0.730838 |
| estro_cond | 0.416657 | 0.208824 | 58.12917 | 1.995256 | 0.050709 |
| c.CESD | -0.00747 | 0.011009 | 113.0593 | -0.67841 | 0.498899 |
| session | 0.174965 | 0.072087 | 57.86967 | 2.427132 | 0.018353 |
| met/val:estro_cond | -0.50854 | 0.230775 | 57.69333 | -2.20362 | 0.031557 |
| val/val:estro_cond | -0.50608 | 0.237022 | 57.64794 | -2.13518 | 0.037008 |

**S28 Table. Forward digit span reduced OC model output – *COMT* coded as a factor.**

lmer(forward ~ COMT*estro_cond + (1|participant) + session + c.CESD, subset = (group==1), data = dataset)

| Model term | Estimate | Std. Error | df | t value | Pr(>\|t\|) |
| --- | --- | --- | --- | --- | --- |
| (Intercept) | 6.614888 | 0.258764 | 53.49629 | 25.56339 | 1.16E-31 |
| met/val | 0.160433 | 0.310753 | 53.31871 | 0.516273 | 0.607799 |
| val/val | -0.34751 | 0.35517 | 53.88412 | -0.97842 | 0.332238 |
| estro_cond | -0.04131 | 0.150734 | 52.77637 | -0.27408 | 0.785095 |
| c.CESD | -0.00041 | 0.013854 | 101.8365 | -0.02953 | 0.976498 |
| session | 0.050528 | 0.073931 | 56.07339 | 0.683446 | 0.49714 |
| met/val:estro_cond | -0.1118 | 0.177274 | 52.52818 | -0.63066 | 0.530996 |
| val/val:estro_cond | 0.104379 | 0.202136 | 52.93377 | 0.516381 | 0.607739 |

**S29 Table. Forward digit span model output – *COMT* coded as numeric.**

lmer(forward ~ group*COMT.effcode*estro_cond + (1|participant) + session + c.CESD, data = dataset)

| Model term | Estimate | Std. Error | df | t value | Pr(>\|t\|) |
| --- | --- | --- | --- | --- | --- |
| (Intercept) | 6.689925 | 0.094063 | 114.8648 | 71.12151 | 8.08E-97 |
| COMT.effcode | -0.10602 | 0.131356 | 116.488 | -0.80712 | 0.421243 |
| estro_cond | -0.02033 | 0.052543 | 114.4421 | -0.38691 | 0.699543 |
| OC_use | -0.08048 | 0.094004 | 114.717 | -0.85617 | 0.393689 |
| c.CESD | -0.00436 | 0.008484 | 227.9139 | -0.51386 | 0.607849 |
| session | 0.10976 | 0.051421 | 114.0396 | 2.134559 | 0.034938 |
| COMT.effcode:estro_cond | -0.05516 | 0.074791 | 113.9126 | -0.73754 | 0.462308 |
| COMT.effcode:OC_use | -0.07383 | 0.130389 | 114.7702 | -0.56623 | 0.572343 |
| estro_cond:OC_use | -0.03981 | 0.052739 | 117.1366 | -0.7549 | 0.451822 |
| COMT.effcode:estro_cond:OC_use | 0.087248 | 0.072126 | 114.1405 | 1.20966 | 0.228909 |

**S30 Table. Backward digit span model output – *COMT* coded as a factor.**

lmer(backward ~ group*COMT*estro_cond + (1|participant) + session + c.CESD, data = dataset)

| Model term | Estimate | Std. Error | df | t value | Pr(>\|t\|) |
| --- | --- | --- | --- | --- | --- |
| (Intercept) | 5.199192 | 0.270649 | 112.3838 | 19.2101 | 2.69E-37 |
| met/val | 0.306205 | 0.310881 | 112.3837 | 0.984961 | 0.326759 |
| val/val | 0.181234 | 0.329418 | 113.1967 | 0.550165 | 0.58329 |
| estro_cond | 0.253812 | 0.140253 | 111.3012 | 1.809669 | 0.073046 |
| group | -0.0863 | 0.270626 | 112.3626 | -0.31888 | 0.750407 |
| c.CESD | -0.00205 | 0.009672 | 220.916 | -0.21222 | 0.832134 |
| session | 0.086209 | 0.056229 | 111.5567 | 1.533174 | 0.128064 |
| met/val:estro_cond | -0.26364 | 0.15914 | 111.2111 | -1.65667 | 0.100406 |
| val/val:estro_cond | -0.30114 | 0.171236 | 111.3124 | -1.75864 | 0.081387 |
| met/val:group | 0.112672 | 0.310858 | 112.3653 | 0.362456 | 0.717692 |
| val/val:group | 0.10946 | 0.328261 | 112.3151 | 0.333455 | 0.739413 |
| estro_cond:group | 0.07826 | 0.137861 | 112.7166 | 0.56767 | 0.571388 |
| met/val:estro_cond:group | -0.11559 | 0.157407 | 111.3841 | -0.73433 | 0.464294 |
| val/val:estro_cond:group | -0.03784 | 0.166169 | 111.7222 | -0.22772 | 0.820283 |

**S31 Table. Backward digit span model output – *COMT* coded as numeric.**

lmer(backward ~ group*COMT.effcode*estro_cond + (1|participant) + session + c.CESD, data = dataset)

| Model term | Estimate | Std. Error | df | t value | Pr(>\|t\|) |
| --- | --- | --- | --- | --- | --- |
| (Intercept) | 5.403032 | 0.111406 | 114.5313 | 48.49866 | 3.44E-78 |
| COMT.effcode | 0.061945 | 0.155479 | 116.1411 | 0.398414 | 0.691057 |
| estro_cond | 0.043599 | 0.057067 | 114.0151 | 0.763996 | 0.446448 |
| group | -0.00589 | 0.111341 | 114.3849 | -0.05289 | 0.957911 |
| c.CESD | -0.00318 | 0.009597 | 225.436 | -0.33127 | 0.740748 |
| session | 0.078395 | 0.055839 | 113.6172 | 1.40394 | 0.163064 |
| COMT.effcode:estro_cond | -0.1416 | 0.081214 | 113.4916 | -1.7436 | 0.083936 |
| COMT.effcode:group | 0.07517 | 0.154434 | 114.4376 | 0.486746 | 0.627369 |
| estro_cond:group | 0.011383 | 0.057337 | 116.6726 | 0.198531 | 0.842975 |
| COMT.effcode:estro_cond:group | -0.01856 | 0.078327 | 113.717 | -0.23692 | 0.813146 |

**S32 Table. AX-CPT PBI accuracy model output – *COMT* coded as a factor.**

lmer(PBI_acc ~ group*COMT*estro_cond + (1|participant) + session + c.CESD, data = acc_PBI_df)

| Model term | Estimate | Std. Error | df | t value | Pr(>\|t\|) |
| --- | --- | --- | --- | --- | --- |
| (Intercept) | 0.030783 | 0.019238 | 110.2114 | 1.600117 | 0.112435 |
| group | 0.013702 | 0.019243 | 110.2463 | 0.712035 | 0.477947 |
| met/val | 0.006103 | 0.022157 | 110.2897 | 0.275456 | 0.783482 |
| val/val | 0.005896 | 0.023596 | 110.8309 | 0.249857 | 0.803161 |
| estro_cond | 0.01337 | 0.018115 | 109.5877 | 0.738047 | 0.462064 |
| session | 0.007719 | 0.007332 | 109.9655 | 1.052874 | 0.294707 |
| c.CESD | -0.00064 | 0.00093 | 191.2956 | -0.68942 | 0.491397 |
| group:met/val | -0.00424 | 0.022148 | 110.2322 | -0.1916 | 0.848412 |
| group:val/val | -0.01328 | 0.023548 | 110.5432 | -0.56392 | 0.573948 |
| group:estro_cond | 0.002566 | 0.017741 | 110.8013 | 0.144623 | 0.885271 |
| met/val:estro_cond | -0.00976 | 0.020574 | 109.5143 | -0.47423 | 0.636282 |
| val/val:estro_cond | 0.011679 | 0.022223 | 109.6964 | 0.525545 | 0.600265 |
| group:met/val:estro_cond | -0.0039 | 0.020351 | 109.6468 | -0.19182 | 0.848239 |
| group:val/val:estro_cond | 0.011823 | 0.021607 | 110.0858 | 0.547201 | 0.585349 |

**S33 Table. AX-CPT PBI accuracy model output – *COMT* coded as numeric.**

lmer(PBI_acc ~ group*COMT. effcode*estro_cond + (1|participant) + session + c.CESD, data = acc_PBI_df)

| Model term | Estimate | Std. Error | df | t value | Pr(>\|t\|) |
| --- | --- | --- | --- | --- | --- |
| (Intercept) | 0.03535 | 0.00795 | 112.3725 | 4.446407 | 2.06E-05 |
| group | 0.008246 | 0.007954 | 112.4611 | 1.036707 | 0.302096 |
| COMT.effcode | 0.002828 | 0.011115 | 113.6027 | 0.254429 | 0.799624 |
| estro_cond | 0.010977 | 0.007433 | 112.1545 | 1.476832 | 0.142523 |
| session | 0.00687 | 0.007279 | 111.9715 | 0.943789 | 0.347309 |
| c.CESD | -0.00067 | 0.000918 | 193.0205 | -0.73346 | 0.464168 |
| group:COMT.effcode | -0.00661 | 0.011073 | 113.0758 | -0.59692 | 0.551755 |
| group:estro_cond | 0.003611 | 0.007417 | 114.6407 | 0.48692 | 0.627245 |
| COMT.effcode:estro_cond | 0.007319 | 0.010554 | 111.9244 | 0.693493 | 0.489437 |
| group:COMT.effcode:estro_cond | 0.005172 | 0.010207 | 112.235 | 0.506696 | 0.613362 |

**S34 Table. AX-CPT PBI reaction time model output – *COMT* coded as a factor.**

lmer(PBI_rt ~ group*COMT*estro_cond + (1|participant) + session + c.CESD, data = rt_PBI_df)

| Model term | Estimate | Std. Error | df | t value | Pr(>\|t\|) |
| --- | --- | --- | --- | --- | --- |
| (Intercept) | 0.038278 | 0.002645 | 110.9554 | 14.47163 | 2.63E-27 |
| group | 0.001055 | 0.002645 | 110.9554 | 0.398879 | 0.690749 |
| met/val | 0.001968 | 0.003045 | 110.9555 | 0.646344 | 0.519391 |
| val/val | 0.003445 | 0.003234 | 111.1127 | 1.065169 | 0.289109 |
| estro_cond | 0.001954 | 0.001382 | 108.3792 | 1.414197 | 0.16017 |
| session | 0.002827 | 0.00056 | 108.5376 | 5.04741 | 1.82E-06 |
| c.CESD | -1.4E-05 | 0.000128 | 108.5475 | -0.10839 | 0.913884 |
| group:met/val | 0.004223 | 0.003045 | 110.9556 | 1.386843 | 0.168269 |
| group:val/val | 0.001603 | 0.003234 | 111.1127 | 0.495599 | 0.621156 |
| group:estro_cond | 0.000762 | 0.001366 | 108.3728 | 0.55802 | 0.577982 |
| met/val:estro_cond | -0.00224 | 0.001569 | 108.3737 | -1.42887 | 0.155918 |
| val/val:estro_cond | -0.00144 | 0.001696 | 108.4571 | -0.85184 | 0.396181 |
| group:met/val:estro_cond | -0.00037 | 0.001553 | 108.3697 | -0.24072 | 0.810228 |
| group:val/val:estro_cond | -0.00179 | 0.001653 | 108.5027 | -1.08457 | 0.280517 |

**S35 Table. AX-CPT PBI reaction time model output – *COMT* coded as numeric.**

lmer(PBI_rt ~ group*COMT.effcode*estro_cond + (1|participant) + session + c.CESD, data = rt_PBI_df)

| AX-CPT PBI reaction time model terms | Estimate | Std. Error | df | t value | Pr(>\|t\|) |
| --- | --- | --- | --- | --- | --- |
| (Intercept) | 0.040051 | 0.001104 | 113.0252 | 36.29023 | 4.04E-64 |
| group | 0.003674 | 0.001104 | 113.0239 | 3.329429 | 0.001176 |
| COMT.effcode | 0.002211 | 0.001532 | 113.2705 | 1.44298 | 0.151786 |
| estro_cond | 0.000402 | 0.00057 | 110.4841 | 0.705491 | 0.481989 |
| session | 0.002811 | 0.000557 | 110.5474 | 5.045224 | 1.79E-06 |
| c.CESD | -2.2E-05 | 0.000128 | 110.542 | -0.1756 | 0.860931 |
| group:COMT.effcode | 0.000243 | 0.001532 | 113.2705 | 0.158736 | 0.87416 |
| group:estro_cond | 0.000191 | 0.000579 | 110.458 | 0.329284 | 0.742565 |
| COMT.effcode:estro_cond | -0.00038 | 0.000807 | 110.5647 | -0.46899 | 0.639998 |
| group:COMT.effcode:estro_cond | -0.00122 | 0.000782 | 110.6434 | -1.56411 | 0.120647 |

**S36 Table. Digit Ordering Task model output – *COMT* coded as a factor.**

lmer(Total_errors ~ group*COMT*estro_cond + (1|participant) + session + c.CESD, data = dataset)

| Model term | Estimate | Std. Error | df | t value | Pr(>\|t\|) |
| --- | --- | --- | --- | --- | --- |
| (Intercept) | 11.10099 | 1.405526 | 108.8105 | 7.898099 | 2.44E-12 |
| met/val | 0.199399 | 1.618094 | 108.8046 | 0.123231 | 0.902152 |
| val/val | 0.875015 | 1.729294 | 109.3179 | 0.505995 | 0.613879 |
| estro_cond | -0.04335 | 0.720104 | 107.897 | -0.0602 | 0.952108 |
| Group | -2.13441 | 1.405569 | 108.8174 | -1.51853 | 0.13178 |
| c.CESD | 0.008339 | 0.054159 | 215.5074 | 0.153981 | 0.877769 |
| session | -0.31955 | 0.294454 | 108.596 | -1.08523 | 0.280222 |
| met/val:estro_cond | -0.19367 | 0.819031 | 107.7269 | -0.23646 | 0.813522 |
| val/val:estro_cond | -0.08083 | 0.886939 | 107.7344 | -0.09113 | 0.927556 |
| met/val:group | 1.523115 | 1.617997 | 108.7911 | 0.941358 | 0.348607 |
| val/val:group | 2.577719 | 1.725087 | 108.7664 | 1.494254 | 0.138005 |
| estro_cond:group | -0.40225 | 0.707839 | 109.5594 | -0.56827 | 0.571013 |
| met/val:estro_cond:group | 0.987295 | 0.808483 | 107.851 | 1.221169 | 0.224685 |
| val/val:estro_cond:group | 1.418688 | 0.862871 | 108.4029 | 1.644148 | 0.103043 |

**S37 Table. Digit Ordering Task model output – *COMT* coded as numeric.**

lmer(Total_errors ~ group*COMT.effcode*estro_cond + (1|participant) + session + c.CESD, data = dataset)

| Model term | Estimate | Std. Error | df | t value | Pr(>\|t\|) |
| --- | --- | --- | --- | --- | --- |
| (Intercept) | 11.4074 | 0.581937 | 110.8482 | 19.60247 | 8.1E-38 |
| COMT.effcode | 0.509099 | 0.816289 | 111.8106 | 0.623675 | 0.534111 |
| estro_cond | -0.16881 | 0.293684 | 110.4643 | -0.57481 | 0.566586 |
| group | -0.7157 | 0.581935 | 110.8473 | -1.22985 | 0.221356 |
| c.CESD | 0.009601 | 0.053541 | 219.6371 | 0.179316 | 0.857855 |
| session | -0.30997 | 0.290112 | 110.6047 | -1.06845 | 0.287645 |
| COMT.effcode:estro_cond | 0.021862 | 0.41995 | 109.7425 | 0.052059 | 0.958576 |
| COMT.effcode:group | 1.215941 | 0.812767 | 110.8205 | 1.49605 | 0.137483 |
| estro_cond:group | 0.457536 | 0.297159 | 114.4076 | 1.539703 | 0.126394 |
| COMT.effcode:estro_cond:group | 0.642339 | 0.407094 | 110.5363 | 1.577864 | 0.117454 |
